# Structural insights of an LCP protein–LytR–from *Streptococcus dysgalactiae subs. dysgalactiae* through biophysical and *in silico* methods

**DOI:** 10.3389/fchem.2024.1379914

**Published:** 2024-08-06

**Authors:** João Paquete-Ferreira, Filipe Freire, Henrique S. Fernandes, Jayaraman Muthukumaran, João Ramos, Joana Bryton, Alejandro Panjkovich, Dmitri Svergun, Marino F. A. Santos, Márcia A. S. Correia, Alexandra R. Fernandes, Maria João Romão, Sérgio F. Sousa, Teresa Santos-Silva

**Affiliations:** ^1^ Associate Laboratory i4HB–Institute for Health and Bioeconomy, NOVA School of Science and Technology, Universidade NOVA de Lisboa, Caparica, Portugal; ^2^ UCIBIO—Applied Molecular Biosciences Unit, Department of Chemistry, NOVA School of Science and Technology, Universidade NOVA de Lisboa, Caparica, Portugal; ^3^ Associate Laboratory i4HB—Institute for Health and Bioeconomy, University of Porto, Porto, Portugal; ^4^ UCIBIO—Applied Molecular Biosciences Unit, Faculty of Medicine, University of Porto, Porto, Portugal; ^5^ European Molecular Biology Laboratory, Hamburg Unit, Deutsches Elektronen-Synchrotron, Hamburg, Germany; ^6^ UCIBIO—Applied Molecular Biosciences Unit, Department of Life Sciences, NOVA School of Science and Technology, Universidade NOVA de Lisboa, Caparica, Portugal

**Keywords:** LytR-CpsA-Psr, X-ray diffraction, SAXS, molecular dynamics, docking, wall teichoic acids, pyrophosphatase

## Abstract

The rise of antibiotic-resistant bacterial strains has become a critical health concern. According to the World Health Organization, the market introduction of new antibiotics is alarmingly sparse, underscoring the need for novel therapeutic targets. The LytR-CpsA-Psr (LCP) family of proteins, which facilitate the insertion of cell wall glycopolymers (CWGPs) like teichoic acids into peptidoglycan, has emerged as a promising target for antibiotic development. LCP proteins are crucial in bacterial adhesion and biofilm formation, making them attractive for disrupting these processes. This study investigated the structural and functional characteristics of the LCP domain of LytR from *Streptococcus dysgalactiae* subsp. dysgalactiae. The protein structure was solved by X-ray Crystallography at 2.80 Å resolution. Small-angle X-ray scattering (SAXS) data were collected to examine potential conformational differences between the free and ligand-bound forms of the LytR LCP domain. Additionally, docking and molecular dynamics (MD) simulations were used to predict the interactions and conversion of ATP to ADP and AMP. Experimental validation of these predictions was performed using malachite green activity assays. The determined structure of the LCP domain revealed a fold highly similar to those of homologous proteins while SAXS data indicated potential conformational differences between the ligand-free and ligand-bound forms, suggesting a more compact conformation during catalysis, upon ligand binding. Docking and MD simulations predicted that the LytR LCP domain could interact with ADP and ATP and catalyze their conversion to AMP. These predictions were experimentally validated by malachite green activity assays, confirming the protein’s functional versatility. The study provides significant insights into the structural features and functional capabilities of the LCP domain of LytR from *S. dysgalactiae* subsp. dysgalactiae. These findings pave the way for designing targeted therapies against antibiotic-resistant bacteria and offer strategies to disrupt bacterial biofilm formation.

## 1 Introduction

The bacterial cell wall is the first line of contact for the cell with the environment. It is responsible for functions related to protection and communication with the surroundings, but it also plays a very important structural role in preserving the cell’s shape and increasing resistance to mechanical stress (Cabeen and Jacobs-Wagner, 2005).

In Gram-positive bacteria, the cell wall is constituted of a thick layer of peptidoglycan decorated with polymers and glycopolymers (Siegel et al., 2016). These modifications confer different properties to the cell wall, affecting porosity, charge, communication, and protection. In fact, some of these polymers are essential for cell viability and pathogenesis (Weidenmaier and Peschel, 2008).

The wall teichoic acids (WTAs) are among these polymers. They make up most of the polymers present in the cell wall, representing up to 60% of its total mass. Unlike their counterpart, lipoteichoic acids (LTAs), WTAs are not anchored to the cell membrane but are covalently attached to the peptidoglycan through a phosphodiester bond between the WTAs and the C6 hydroxyl group of the N-Acetylmuramic acid (MurNAc) residues of the peptidoglycan (Brown et al., 2013). Nevertheless, both types of teichoic acids, WTAs and LTAs, share a general structure consisting of a linkage unit and a long main chain. In the case of the WTAs, the linkage unit holds an N-Acetylmannosamine (ManNAc) (β1→4) N-Acetylglucosamine (GlnNAc)-1-phosphate and a small chain of glycerol 3-phosphate, whereas the LTAs linkage unit consists of a glycolipid that varies among the different types of LTAs. The main chain of both glycopolymers contains different repeating units, generally glycerol 3-phosphate or ribitol 5-phosphate, that can be further decorated (Brown et al., 2013; Chapot-Chartier and Kulakauskas, 2014; Schneewind and Missiakas, 2014).

The structural similarity between WTAs and LTAs leads to some overlapping roles, even if other features are specific to the WTA (Swoboda et al., 2010). One of the important functions of WTAs is the binding of positively charged ions (e.g., Ca^2+^ and Mg^2+^). Since WTAs are more exposed than LTAs, there are numerous negatively charged phosphate groups available for ionic interactions, allowing the cell to keep a storage of metals near the surface. This also creates localized pH changes through modulation of the proton binding capacity (Biswas et al., 2012; Brown et al., 2013). Another important function of the WTAs is related to the cross-linking of the peptidoglycan which occurs after WTAs attachment. It has been suggested that their presence guides the cross-linking process, either by playing scaffolding roles or contributing to stereochemical hindrances (Campbell et al., 2011). This same scaffolding role of the WTAs seems to be important in cell division and morphology (Biswas et al., 2012). Schaefer and colleagues showed that non-cross-linked peptidoglycan is a suitable substrate for WTAs modification and that WTAs transfer prior to cross-linking suggests that these molecules might have a regulatory role in the cell wall maturation process (Schaefer et al., 2018). However, this is still a debatable topic and further research is necessary (Brown et al., 2013). WTAs have also been considered important for the pathogenesis of some bacteria, especially when these polymers are further modified by D-alanylation, as they allow for overcoming the host-defense mechanisms or modulating the host-tissue adhesion during biofilm formation. (Gross et al., 2001), Despite the crucial role of WTAs, they are not considered virulence factors. This is because their absence leads to non-viable or highly defective cells, establishing their biosynthetic pathway as very interesting drug targets for the development of new antibiotics (Brown et al., 2013). Several studies suggest that WTAs depletion is an effective strategy for eliminating bacteria and inhibiting biofilm formation. Brown and colleagues showed that methicillin-resistant *Staphylococcus aureus* (MRSA) mutated strains regain their susceptibility towards methicillin (Campbell et al., 2011; Brown et al., 2012).

Several antibiotics target the cell wall biosynthesis pathway in bacteria, namely, by inhibiting peptidoglycan biosynthesis. However, the increasing number of bacterial strains resistant to common antibiotics urges finding new targets (Årdal et al., 2019; Miethke et al., 2021; Varela et al., 2021). The LytR-CpsA-Psr (LCP) (LytR - autolysin regulator; CpsA–capsule-associated protein A and Psr–penicillin-binding protein regulator) is a family of enzymes responsible for the last step of the assembly of WTA and other cell wall glycopolymers. It has been considered as important drug targets, but so far, no efficient inhibitors have been described (Kawai et al., 2011).

In this work, we studied the LytR protein from the Gram-positive bacterium *Streptococcus dysgalactiae subsp. dysgalactiae* (SDSD), a known animal pathogen and one of the leading causes of bovine mastitis and fish streptococcosis (Nomoto et al., 2004; Alves-Barroco et al., 2019a). These diseases lead to serious consequences and losses for the dairy and aquaculture industries (Abdelsalam et al., 2013). Besides, SDSD can form biofilms making it very difficult to eliminate, since in that growth phenotype, bacterial are a lot less susceptible to antimicrobial agents. SDSD was shown to also infect humans (Park et al., 2012; Roma-Rodrigues et al., 2016; Alves-Barroco et al., 2019b; Alves-Barroco et al., 2022).

LytR and other members of the LCP family have been described as pyrophosphatases. They cleave the pyrophosphate bond in glycopolymers precursors. These precursors contain several undecaprenyl units, the pyrophosphate group and the glycopolymer that is going to be transferred to the peptidoglycan, usually to the MurNAc residues. This process occurs in the outer layer of the cell wall. Besides very complex and large polymers, these enzymes can also bind shorter molecules containing only the hydrophobic undecaprenyl units (decaprenyl, octaprenyl and geranylgeranyl) and phosphate/pyrophosphate groups (Kawai et al., 2011; Eberhardt et al., 2012). In fact, these simpler substrates are commonly used in activity assays although their low solubility in aqueous solutions is a great challenge.

LCPs can be found in virtually all Gram-positive bacteria, and commonly, with several enzymes per organism (up to 11 as in *Streptococcus coelicor*) (Hübscher et al., 2008). The presence of these different members is relevant for functional redundancy purposes and usually, only the knock-out of all the LCP proteins will lead to non-viable cells (Kawai et al., 2011).

The LCP proteins have a quite variable topology. Besides the LCP domain, that is extracellular and structurally very conserved, these proteins often possess accessory domains of unknown function. Accessory domains can be present both in the N- or C-terminus relative to the LCP domain and can be intracellular or extracellular. For example, the Wzg protein from *Streptococcus pneuomoniae* has an extracellular accessory domain in the N-terminus, while LytR from SDSD has a similar extracellular domain but in the C-terminus and Lcp2 from SDSD has the accessory domain in the N-terminus, intracellularly (Kawai et al., 2011). Since these enzymes operate on the outer side of the cell wall, they possess anchoring helices that attach the LCP domain to the membrane, connecting it to the auxiliary domain when this is present. According to available data, LCP proteins commonly have one transmembrane helix, but these can go up to three as in the CpsA proteins (Hübscher et al., 2008).

In this study, our goal is to investigate the structure and function of the LytR protein from *Streptococcus* dysgalactiae subsp. dysgalactiae (SDSD) and its implications in the biosynthesis of wall teichoic acids. Through a combination of crystallography, X-ray scattering, molecular dynamics simulations, docking, and activity assays, we aim to elucidate the molecular mechanisms underlying the enzymatic activity of LytR and its potential as a target for novel antibiotics.

## 2 Materials and methods

### 2.1 Plasmid construction

To produce the LytR protein from *S. dysgalactiae subs. dysgalactiae*, a DNA fragment coding the amino acid residues 48–342 was amplified from genomic DNA (VSD9 strain). The amplified fragment was then cloned into a pET21c (+) vector (Novagene) into the NdeI and XhoI restriction sites. The primers used can be found in Table 1 of Supplementary Material. The region comprising residues 1–47 corresponds to the protein’s signal peptide and was not included, as well as residues 342–422 which are predicted to be highly flexible and disordered.

**TABLE 1:** X-ray Data collection and Refinement statistics of LytR LCP domain from *Streptococcus dysgalactiae* subsp. *dysgalactaie*.

PDB ID	8QTY
**Space Group**	I 4 2 2
**Unit Cell**	
(a, b, c), Å	141.27 141.27 133.74
Molecules per Asymmetric Unit	1
Matthews Coefficient	4.98
Solvent Content, %	75
**Data Collection**	
Distance to detector, cm	34.091
Wavelength, Å	0.966
Collected Images	747
Processed Images	747
Resolution Range, Å	48.56–2.80 (2.95–2.80)
R_pim_	0.049 (0.885)
Completeness, %	99.9 (99.9)
<I/*σ*(I)>	15.5 (1.9)
Half-set correlation coefficient, CC_1/2_	0.999 (0.666)
Observed Reflections	136,196 (20,406)
Unique Reflections	16,955 (2,436)
Multiplicity	8.0 (8.4)
**Refinement**	
R_work_/R_free_, %	27.13/29.27
R.M.S.D. Bond Lengths, Å	0.017
R.M.S.D. Bond Angles, °	1.968
**Ramachandran Plot**	
Favored/Disallowed, %	90.6/0.0

### 2.2 Protein expression and purification

*E. coli* BL21 (DE3) cells were transformed with the plasmid DNA and the cells were grown in LB medium supplemented with ampicillin (100 μg/mL), at 37°C. Once the optical density reached 0.5–0.8, protein expression was induced with 1 mM isopropyl β-D-1-thiogalactopyranoside (IPTG), for 5 h, at 30°C. Cells were collected through centrifugation (7477 x g, 15 min at 4°C), resuspended in the lysis buffer (10 mM Na_2_PO_4_ pH 7.2, 100 mM NaCl, 5 mM MgCl_2_, 10 mM imidazole) and lysed by sonication using 10 × 1 min cycles (80% amplitude, 0.5 cycles) (UP100H MS7, Hielscher Ultrasonics). The lysate was clarified through centrifugation (12,857 x g, 60 min at 4°C). LCP domain was purified using an immobilized metal-affinity chromatography (IMAC) column HisTrap HP 5 mL (Cytiva). The protein was eluted using a 30–500 mM linear imidazole gradient. Fractions containing the protein of interest were polled together, and the buffer was exchanged using HiTrap desalting columns (Cytiva) to 10 mM Na_2_PO_4_ pH 7.2, 500 mM NaCl, and 5 mM MgCl_2_, which allowed increasing NaCl concentration and removing the imidazole. The sample was later concentrated and loaded into a size exclusion chromatography (SEC) column Superdex 75 10/300 GL (Cytiva) equilibrated with the desalting buffer. The fractions containing pure LytR LCP domain were pooled together, concentrated, and stored at −20°C.

### 2.3 Crystallization and structure determination

Protein crystals were obtained by sparse-matrix screening (JBScreen Classic 1 to 4 and JBScreen Classic 5 to 8) using an Oryx 8 crystallization robot and two protein concentrations (100 and 200 mg/mL). Crystals appeared in different conditions using the sitting drop vapor diffusion technique, but the best diffracting ones were found with 2.2 M ammonium sulphate and 20% (v/v) glycerol. The crystals grew at a maximum size of 0.06 × 0.06 × 0.06 mm in 4 days, were cryo-protected in parathone and flash-frozen in liquid N_2_. Diffraction data were collected at the European Synchrotron Radiation Facility (ESRF, Grenoble, MASSIF-1 beamline) up to 2.80 Å resolution, processed with XDS, and merged and scaled with AIMLESS (Kabsch, 2010; Evans and Murshudov, 2013). The structure was solved by molecular replacement with MR BUMP (from the CCP4 package) (Keegan and Winn, 2008). using 3OKZ as search model. The refinement was performed with REFMAC5 (from the CCP4 package) (Murshudov et al., 2011). The manual construction and visual inspection of the model were done with COOT (data collection and refinement statistics in Table 1). (Emsley et al., 2010; Kovalevskiy et al., 2016) The resulting model was deposited in the Protein Data Bank (PDB) with the accession code 8QTY.

### 2.4 Small angle X-ray scattering (SAXS)

SAXS experiments were performed at the ESRF, beamline BM29. Two samples of protein at 7.2 mg/mL in 50 mM HEPES pH 8.0, 150 mM NaCl, 5 mM MgCl_2_ and 40% ethylene glycol were prepared in the absence (S1) and in the presence (S2) of 540 uM geranylgeranyl pyrophosphate. Scattering data was collected as intensity *I*(*s*) *versus s*, where *s* = 4πsinθ/λ nm^−1^, 2θ is the scattering angle, and λ is the X-ray wavelength, 0.124 nm. Data processing, reduction and primary analysis were performed with the program PRIMUS (Konarev et al., 2003). This allowed us to obtain the overall parameters (radius of gyration, R_g_, forward scattering, I_0_) and to compute the distance distribution function p(r) using GNOM (Svergun, 1992). The overall parameters of the data collection and analysis are summarized in Table 2. The scattering from the crystal structure and from the models was computed and compared with the experimental data using the program CRYSOL (Svergun et al., 1995). The high resolution models with added flexible chains were refined against the scattering data by SREFLEX (Panjkovich and Svergun, 2016).

**TABLE 2:** SAXS sample and analysis for LytR LCP domain.

(a) Sample details		
Organism	*Streptococcus dysgalactiae* subsp. *dysgalactaie*	
Source	LytR protein, *E. coli* (BL21) recombinant expression	
Scattering particle composition	S1 (SASDTH2)	S2 (SASDTG2)
Protein	LytR LCP domain	
Ligand		Geranylgeranyl pyrophosphate
Stoichiometry of components		1:2
Solvent composition	50 mM HEPES pH 8.0, 150 mM NaCl and 5 mM MgCl_2_	
Sample concentration (mg/mL)	3.05	2.19

(b) SAS data collection		
	DESY P12	ESRF BM29
Data-acquisition/reduction software	*BECQUEREL*	*BSXCuBE*
Source/instrument description	PETRA III U29 undulator, Pilatus 6M	2 Pole Wiggler, Pilatus3 2M in-vac
Mesaured q-range (q_min_ - q_max_) (Å^-1^)	0.0022–0.482	0.0042–0.522
Exposure time (s), No. of exposures	0.045, 15 frames	10 frames

(c) SAS-derived structural parameters		
Method(s)/software	S1 (SASDTH2)	S2 (SASDTG2)
Guinier analysis		
I (0) ± 𝜎 (cm-1)	11.42 ± 0.069	7.41 ± 0.063
Rg ± 𝜎(nm)	2.3 ± 0.02	2.45 ± 0.03
qRg range (datapoint range	0.31–1.13 (16–97)	0.36–1.28 (12–94)
Linear fit assessment (AUTORG fidelity)	0.55	0.99
PDDF/P(r) analysis		
I (0) ± 𝜎 (cm-1)	9.17 ± 0.14	7.37 ± 0.054
Rg ± 𝜎(nm)	2.42 ± 0.0068	2.43 ± 0.017
dmax (nm)	9.0	8.0
q-range (nm^-1^)	0.19–3.37	0.014–0.326
p(r) reciprocal-space fit	0.78	0.69

### 2.5 Molecular dynamics

A preparation step of the crystal structure was required prior to the Molecular Dynamics (MD) simulations. In this step, it was necessary to add the missing loop (G70 to Q77) with Modeler (version 9.23) (Fiser et al., 2000). The protonation state of all residues at the physiological pH of 7.4 was estimated in the PlayMolecule ProteinPrepare web server (Martínez-Rosell et al., 2017). The addition of an Mg^2+^ ion was performed using VMD and the molUP plugin, available through the VMD Store. (S. Fernandes et al., 2018; 2019). The ligand LIIa-WTA was added to the protein by superposition with the deposited protein structure 6MPS and the remaining ligands were obtained based on this one, using also VMD (Humphrey et al., 1996). MD simulations were performed with the Amber software (version 20) using forcefield amber14sb (recommended forcefield for protein simulations within the amber forcefield family), in a 12 Å cubic box that was filled with TIP3P water molecules (Maier et al., 2015; Case et al., 2020). The utilization of TIP3P water molecules reduces the required computational power and is a regular choice when using the forcefield amber14sb. Parametrization of the several used ligands (LIIa-WTA, prenyl-11-g3p-20, prenyl-11-g3p-5, prenyl-6-g3p-5, prenyl-3-g3p-5, prenyl-2-g3p-5 and prenyl-1-g3p-5–Figure 6) was done with Antechamber using the second generation of the General Amber Force Field (GAFF2) (Wang et al., 2004). To obtain the partial atomic charges for these ligands, the Restrained ElectroStatic Potential (RESP) charge derivation protocol was used (Sun et al., 1992). These charges were calculated from the respective optimized structure at the Hartree-Fock level of theory with the 6–31G(d) basis set (i.e., HF/6–31G(d)). The geometry optimization was performed using Gaussian09. For ADP, the parameters were obtained from the Bryce parameters database (Meagher et al., 2003).

Four minimization steps were applied to remove clashes, followed by two equilibration steps and a final production run. The minimization procedure consisted of four stages. In each minimization stage, the first half of the total steps use the steepest descent method, changing thereafter to the conjugate gradient method. Firstly, only the water molecules were minimized (5,000 steps), with Cartesian positional restraints with a weight of 50 kcal mol^-1^. Å^2^ applied on all other atoms. Secondly, all hydrogen atoms were minimized (5,000 steps), with the same restraint weight applied to all non-hydrogen atoms. Thirdly, all non-backbone atoms in the system were minimized (5,000 steps), and the same restraint weight was applied to all protein backbone atoms. Lastly, all system atoms were minimized with no restraints (10,000 steps).

The two 50 ps equilibration steps consisted of: heating the system to 310 K using a Langevin thermostat at constant volume (NVT ensemble); and equilibration of the density of the system at 310 K. Lastly, the 1,000 ns production run was performed in an NPT ensemble with a temperature of 310 K and 1 bar pressure. A time step of 2 fs was used, and the SHAKE algorithm was applied to constrain the bonds involving hydrogen atoms. Analysis of the molecular dynamics was performed using cpptraj and molecular visualisation and inspection were done with VMD (Roe and Cheatham, 2013).

The K-means clustering method was used to analyze the trajectory, group, and characterize the different intermediate states into 3 main clusters of conformations based on the Root Mean Square Deviation (R.M.S.D.) of all non-hydrogen atoms as a measure of similarity. Representative average structures for each of these 3 clusters were determined.

Simulations were repeated with a simple neutralized simulation box in the presence of specific ionic strength, with 500 mM of NaCl, and in the absence of a Mg^2+^ bound to the protein. Besides, simulations were also repeated in the presence of Mg^2+^ and LipidII-WTA (a truncated precursor of the WTA bound to the lipidic precursor) and later extended to several ligands with different numbers of prenyl units in the hydrophobic chain. Additionally, simulations in the presence of a Mg^2+^ ion and ADP or ATP were also performed. In Table 2 from Supplementary Material there is a description of the conditions for all the MD simulations performed.

### 2.6 Docking

The target for the docking was obtained from the major cluster of the molecular dynamics in the presence of LIIa-WTA, and a Mg^2+^ ion. The ligand was removed, and a version with the hydrophobic region trimmed, the 2 prenyl units, was used to define the binding region; the Mg^2+^ was kept in. The structures of ADP, ATP and the other validation molecules (LIIa-WTA, LI-WTA, octaprenyl-pyrophosphate-GlcNAc and octaprenyl diphosphate) were obtained from the Protein Data Bank (PDB) in sdf format, and using OpenBabel, the protonation was defined. Datawarrior was used to determine some descriptors and chemical properties from the chemical structures, important later for the analysis (Sander et al., 2015). The GOLD (version 5.8) software was used to perform the docking, employing all available scoring functions in GOLD as all shown to represent well the binding of known substrates to these proteins (Jones and Willett, 1995; Jones et al., 1997). 100 GA runs were performed, with a binding region based on a radius of 10 Å from a cavity file, centered on the coordinates of the reference ligand. Visual inspection was performed in Pymol (Schrödinger, 2024). Further data processing was done in Datawarrior and Microsoft Excel datasheets.

### 2.7 Activity assay - malachite green

The malachite green assay was used to determine the amount of released Pi from the substrate, thus determining the pyrophosphatase activity of the protein. Protein at a concentration of 35.5 µM, in 50 mM HEPES pH 8.0, 150 mM NaCl, and 5 mM MgCl_2_ and ADP and ATP at 750 μM, in the same buffer, were used. Two time-points were collected, the first one measured after mixing the protein with ADP or ATP and the second after incubating the mixture overnight, at 37°C. Before the assay, a calibration curve with serial dilutions of Na_2_HPO_4_ was done. The assay was adapted by Tom Duncan from (Lanzetta et al., 1979) as follows: 25 µL of the sample were mixed with 100 µL of a solution containing a mixture of 1 volume of 0.045% (w/v) malachite green oxalate, 3 volumes of 4.2% (w/v) sodium molybdate in 4 N HCl and 5 mL of 2% (w/v) Triton-X100 per each 0.1 mL of solution; after 2 min, 12.5 µL of a 34% (w/v) sodium citrate solution were added followed by another incubation at room temperature for 5 min before measuring the absorbance at 650 nm in a microplate reader. Triplicates were performed for the calibration curve and the protein-ADP assays.

## 3 Results

### 3.1 LytR overall structure

The structure of the LCP domain of LytR, comprising residues S48 to S342, was solved by molecular replacement using gbs0355 from *Streptococcus agalactiae* (PDB ID: 3OKZ) as a model (sequence identity 75.4%) at a resolution of 2.8 Å (Data collection and refinement statistics are available in Table 1). Analysis of the electron density maps suggests the presence of three sulphate ions at the surface of the protein. No electron density was observed at the active site that could correspond to bound substrates. The regions S48-Q56, G70-Q77, and N335-S342 are highly disordered, with no interpretable electron density in the 2Fo-Fc map. For this reason, these residues were not included in the final model.

**FIGURE 1:**
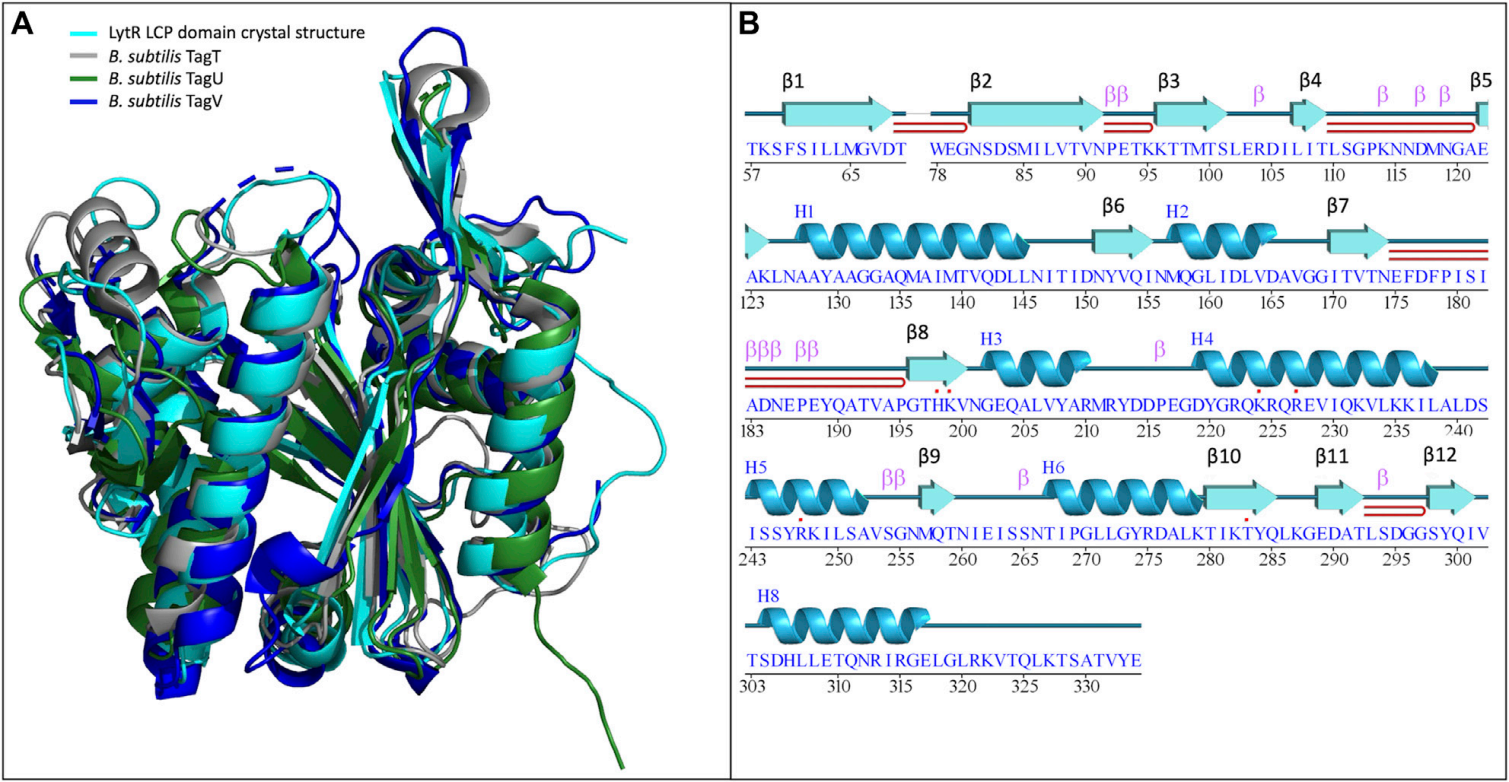
**(A)-** superposition of the LytR LCP domain crystal structure (represented in light blue) with the deposited structures of *B. subtilis* TagT (represented in grey), TagU (represented in dark green) and TagV (represented in dark blue) (PDB IDs: 8QTY. 6UF5, 6UF6 and 6UF3, respectively) (Li et al., 2020); **(B)-** schematic representation of the secondary structure elements identified in the crystal structure of the LytR LCP domain.

The structure is composed of 8 α-helices alternated with 12 β-sheets in a 3-layer (αβα) sandwich architecture (Figure 1). The characteristic central six-stranded β-sheet is sandwiched between the α-helices and the double-stranded antiparallel β-sheets (β4-β5, β7-β8 and β11-β12). The overall structure determined was compared with those of other LCP domains (sequence identity and RMSD of the superpositions are in Table 3; Figure 1). The results of the comparison are align with what is described in the literature and show that, despite the low sequence identity among the proteins of the LCP family, the structural similarities are striking.

**TABLE 3:** Comparison of sequence identity and root mean square deviation (R.M.S.D.) values of LytR LCP domain with other deposited models.

Protein	PDB ID	Sequence identity (with LytR)	R.M.S.D. free form vs*.* ligand bound (aligned C_α_)	R.M.S.D. towards LytR (aligned C_α_)
TagT from *B. subtilis*	6UF5 (free form) (Li et al., 2020)	25.6%	-	1.12 Å (168/223)
	6MPS (bound to LIIa-WTA) (Schaefer et al., 2018)		0.44 Å (228/245)	1.06 Å (171/252)
	6MPT (bound to LI-WTA) (Schaefer et al., 2018)		0.27 Å (210/245)	1.16 Å (177/230)
	4DE9 (bound to octaprenyl phosphate) (Eberhardt et al., 2012)		0.30 Å (217/217)	1.15 Å (164/269)
TagU from *B. subtilis*	6UF6 (Li et al., 2020)	29.7%	-	2.67 Å (169/201)
TagV from B. *subtilis*	6UF3 (Li et al., 2020)	29.0%	-	1.65 Å (178/216)
Wzg from *S. pneumoniae*	2XXP (bound to octaprenyl phosphate) (Kawai et al., 2011)	21.7%	-	1.20 Å (149/202)
	4DE8 (R267A) (bound to octaprenyl phosphate) (Eberhardt et al., 2012)		-	1.23 Å (149/202)
	3TFL (bound to octaprenyl diphosphate) (Kawai et al., 2011)		-	1.25 Å (149/202)
	2XXQ (R267A) (bound to octaprenyl diphosphate) (Kawai et al., 2011)		-	1.28 Å (149/202)
LcpA from *S. aureus*	6UEX (bound to octaprenyl-pyrophosphate-GlcNAc) (Li et al., 2020)	30.4%	-	1.71 Å (169/219)

**FIGURE 2:**
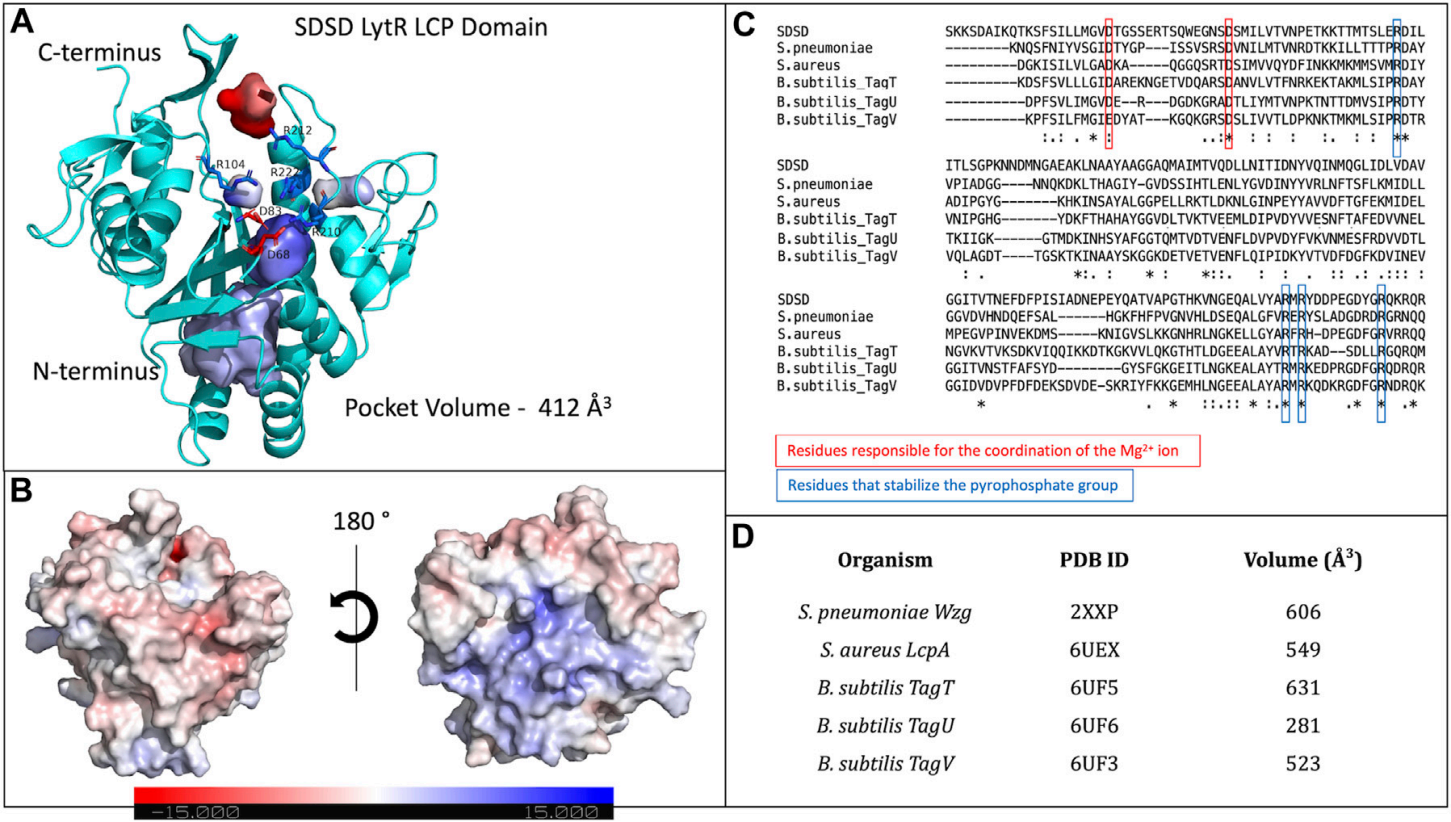
**(A)**- representation of the LytR LCP domain crystal structure determined at 2.8 Å resolution (PDB ID 8QTY). The surface of the pocket is shown in purple and in red. The arginine residues (dark blue) that stabilize the pyrophosphate group and the aspartate residues (red), that coordinate the Mg^2+^ ion (not present in the structure), are represented as stick; **(B)**- electrostatic surface of the LytR LCP domain in two orientations; **(C)**- alignment of some LCP sequences from deposited structures in the PDB. The conserved residues present in the catalytic site are represented: in red, for the residues responsible for coordination of the Mg^2+^ ion, and in dark blue, for the arginine residues that stabilize the pyrophosphate group; **(D)**- pocket volumes of the LCP proteins present in the alignment and their respective PDB IDs (Kawai et al., 2011; Li et al., 2020).

LytR LCP domain contains a long and narrow pocket located between the central β-strand and the α-helices 3 to 7, near the N-terminus. This pocket is composed of several different hydrophobic residues (F60, V153, L160, A209, I261 and Y274), that point their side chains towards the pocket. Polar residues (M99, M157, M257, Q226, S84, T97, T259, and N156) are also present, with their side chains towards the pocket, except for N156. The pocket is highly accessible and has a solvent-accessible surface area of 547.75 Å^2^ (values were calculated using Computed Atlas of Surface Topography of proteins - CASTp) (Tian et al., 2018). The presence of this pocket is extremely conserved in the LCP family of proteins corresponding to the binding site of the undecaprenyl units - the hydrophobic section - of the physiological substrates. For LytR LCP domain, the pocket’s volume is 412 Å^3^ (Figure 2).

The binding site of LCP proteins is composed of conserved positive and negatively charged residues: two aspartates (D68 and D83 for SDSD LytR) coordinate the Mg^2+^ cofactor and are present in all members of the family identified so far, except for just two cases. In the *B. subtilis* TagV protein (PDB IDs 3NXH and 6UF3) one of the aspartates is replaced by a glutamate, and in *E. siraeum* LCP protein (EUBSIR_01389–PDB ID 4OBM), only one of the aspartates is present with the other residue replaced by an alanine residue. The positive charges come from four conserved arginines (R154, R210, R212 and R222 for SDSD LytR) that are expected to interact with the pyrophosphate group present in the native substrates (Figure 2). In the case of the *Eubacterium siraeum* LCP protein, the four conserved arginines are replaced by three proline and one threonine residues.

Crystal structures of wildtype Wzg protein and its R267A mutant from *Streptococcus pneumoniae* show high isomorphism with an R.M.S.D. of 0.14 Å upon superposition of 321 aligned Cα out of 380 (Table 3, Supplementary Material), PDB codes are 3TFL and 2XXP for wildtype Wzg, and 2XXQ and 4DE8 for R267A Wzg) (Kawai et al., 2011; Eberhardt et al., 2012). The presence of substrates sitting in the substrate pocket of R267A suggests that mutating this arginine does not impact the protein’s binding ability. Further studies are necessary to understand whether catalysis is affected and if the same effect is observed when other or several arginine residues are mutated at the same time since activity was not measured by the authors,.

With the wide range of available crystal structures of this family of proteins, we conducted a structural comparison between models obtained in the presence and absence of 5 ligands (Table 4): the substrate analogs octaprenyl-diphosphate, Lipid-II-WTA (LII-WTA), Lipid-I-WTA (LI-a-WTA) and octaprenyl-pyrophosphate-GlcNAc and the reaction product decaprenyl-phosphate. The structural resemblance between free and ligand-bound forms (substrate or reaction product) is very high, as observed by the low R.M.S.D. values of the superpositions (Table 3). This suggests that the protein in the crystal forms deposited so far adopts the same conformation regardless of the presence of ligands in the active site. To study the protein structure in solution we used SAXS.

**TABLE 4:** Results of the different scoring functions available in GOLD.

Molecule	Structure	Score				Score normalized			
		PLP	ASP	Chemscore	Goldscore	PLP	ASP	Chemscore	Goldscore
ADP	9 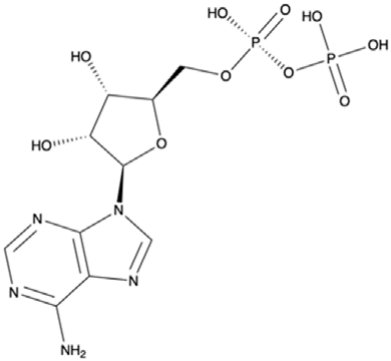	94.54	43.15	16.42	90.10	3.50	1.60	0.61	3.34
ATP	10 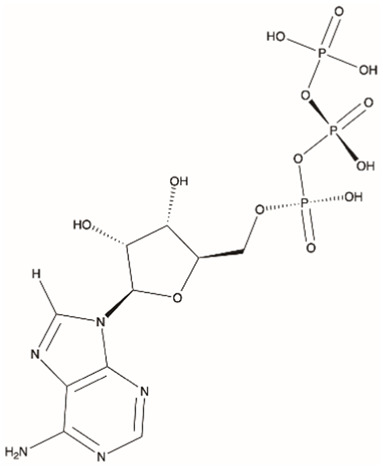	106.68	51.19	17.69	98.11	3.44	1.65	0.57	3.16
LIIa-WTA	2 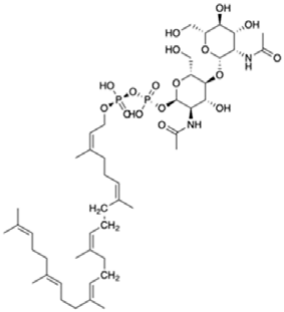	152.52	55.57	45.67	114.53	2.28	0.83	0.68	1.71
LI-WTA	11 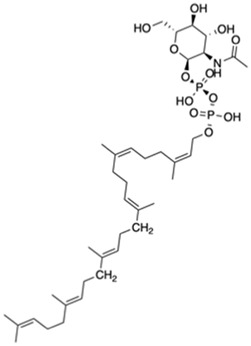	144.34	47.99	48.84	127.26	2.72	0.91	0.92	2.40
Octaprenyl-pyrophosphate-GlcNAc	12 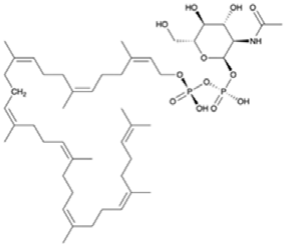	145.61	43.38	50.19	110.80	2.31	0.69	0.80	1.76
Octaprenyl diphosphate	13 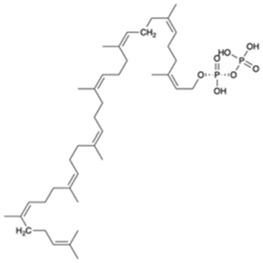	133.59	41.61	49.27	124.18	2.73	0.85	1.01	2.53
Fisetin	14 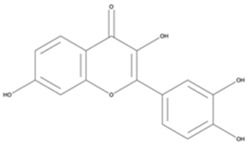	63.41	30.05	27.44	59.46	3.02	1.43	1.31	2.83
Ellagic acid	15 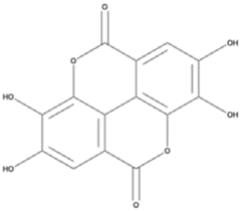	44.83	22.65	22.14	53.83	2.04	1.03	1.01	2.45

### 3.2 Small angle X-ray scattering

**FIGURE 3:**
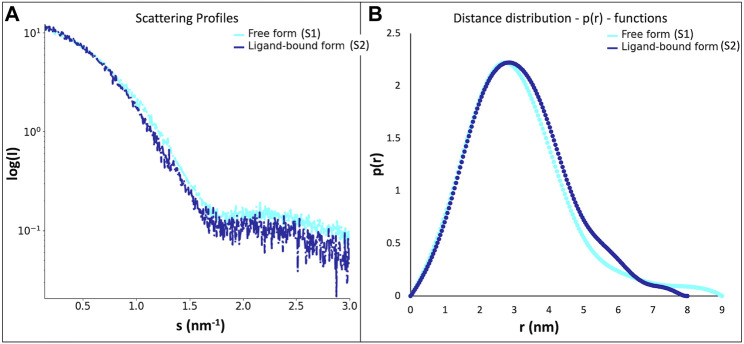
**(A)**- scattering profiles of the LytR LCP domain free form (S1, in light blue) and ligand-bound form (S2, in dark blue); **(B)**- distance distribution functions–p(r)–of the LytR LCP domain free form (S1, in light blue) and ligand-bound form (S2, in dark blue).

SAXS data (Table 2; Figure 3) was collected in batch mode, with protein in the absence (S1) and in the presence (S2) of geranylgeranyl pyrophosphate, a substrate analogue of this protein family that mimics the hydrophobic part of the physiological substrates. Data reduction and initial analysis revealed the presence of concentration effects on the value of R_g_ for all the experiments performed and allowed the determination of R_g_ and I_0_ values. The pair-distance distribution functions (p(r)) were calculated using the scattering at high angles of the highest concentration curves merged with the scattering at low angles of the lowest concentration curves; the p(r) functions (Figure 3B) yielded the D_max_ values.

The experiments performed in the absence and presence geranylgeranyl pyrophosphate revealed some changes in the p(r) profiles (Figure 3). The radius of gyration (about 2.4 nm) remained practically unchanged upon ligand addition. However, a decrease in the D_max_ value by about 0.9 nm was observed in the presence of geranylgeranyl pyrophosphate, suggesting that the protein in solution adopts a somewhat more closed conformation upon ligand interaction.

**FIGURE 4:**
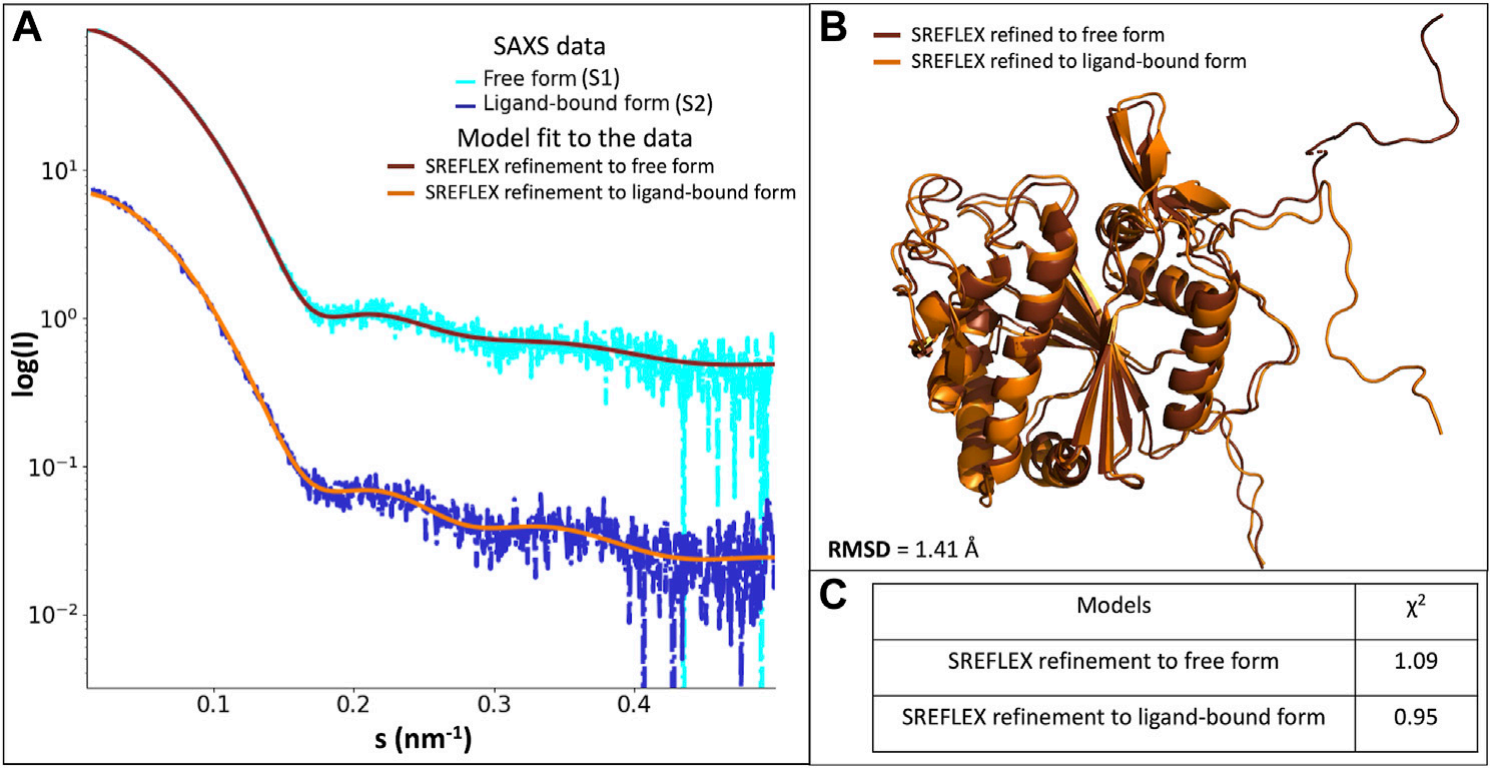
**(A)-** scattering profiles of the scattering profiles of the LytR LCP domain free form (S1, in light blue) fitted with the SREFLEX refined model (brown), and ligand-bound form (S2, in dark blue) fitted with the SREFLEX refined model (orange). **(B)-** superposition of the SREFLEX refined models to the S1 (brown) and to S2 (orange). **(C)**- χ2 values of the fitted SREFLEX models.

To obtain a more detailed assessment of the protein conformation and flexibility in solution, we generated a tentative model of full-length LytR LCP domain based on the crystal structure. We added the missing portions S48-Q56, G70-Q77, and N335-S342 using MODELER. This model yielded a good agreement with the experimental SAXS data in the presence of a ligand (discrepancy χ2 = 1.12), but a poor fit in the absence of a ligand (discrepancy χ2 = 1.91). Further refinement with SREFLEX provided models yielding an improved agreement to the experimental data. In the presence of ligand, the refined model (R.M.S.D. of 2.3 Å to the initial one) had a discrepancy of χ2 = 0.95 to the experimental curve with the ligand. The model in the absence of ligand had an RMSD of 4.8 Å to the initial model, providing a discrepancy of χ2 = 1.09 to the experimental data without ligand. The fits of the refined models to the SAXS data are very good as displayed in Figure 4. Figure 1, from Supplementary Material, shows the comparison between the SREFLEX derived models and the LCP domain full-length.

The SAXS analysis confirmed that the overall crystal structure of LytR is largely preserved in solution and the missing portions in the crystal display significant flexibility. Tentative conformations of these portions were visualized by fitting the SAXS data collected from the full-length domain. Likely, the differences between the native protein and the one with bound geranylgeranyl pyrophosphate can be largely attributed to the changes in the flexible portions of LytR.

### 3.3 Molecular dynamics

**FIGURE 5:**
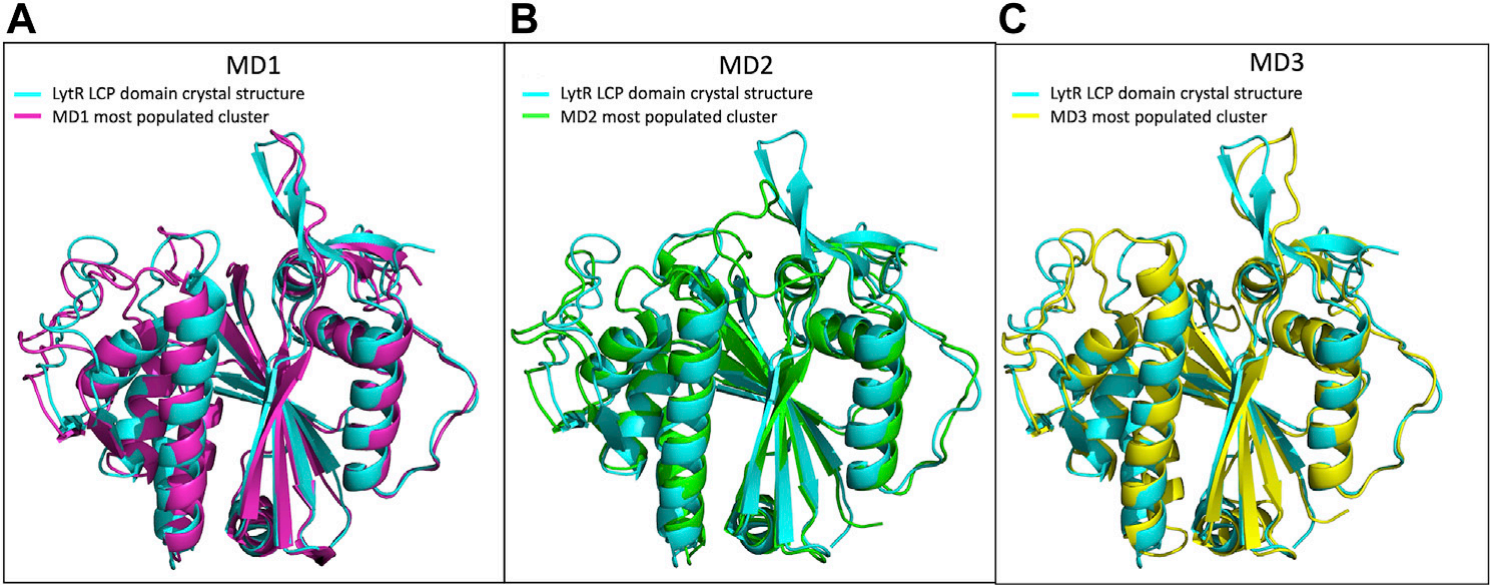
Superposition of the LytR LCP domain crystal structure (PDB ID 8QTY) with the structures representing the most populated clusters of MD1, MD2 and MD3. **(A)**- Superposition of the LytR LCP domain crystal structure (light blue) with the structure representing the most populated cluster of MD1 (pink); **(B)**- superposition of the LytR LCP domain crystal structure (light blue) with the structure representing the most populated cluster of MD2 (green); **(C)**- superposition of the LytR LCP domain crystal structure (light blue) with the structure representing the most populated cluster of MD3 (yellow).

We performed molecular dynamics simulations for the LytR LCP domain in the absence of ionic strength and in the presence of an Mg^2+^ ion bound to the protein. We used the crystal structure described here as the initial model. The results allowed us to determine the main modes of vibration of the protein and their distribution in conditions that mimic the experimental ones. This helped identifying the most affected regions upon visual inspection. Overall, and accordingly with what was expected, loops were the regions with the highest movement. Cluster analysis allowed us to identify three main clusters, with a distribution of 87.8%, 10.6% and 1.6%, respectively. The overall conformation of the protein is the same in all clusters and the R.M.S.D. of the superpositions ranges from 0.758 to 1.491 Å (Table 4; Supplementary Material). To understand if the ionic strength alone could alter the conformation of the LytR LCP domain, we conducted another MD simulation of the LytR LCP domain in the presence of 500 mM NaCl and the absence of the Mg^2+^ ion bound to the protein. For this MD simulation, the cluster analysis revealed a distribution of 40.6%, 22.3% and 13.4% with the R.M.S.D. between the three determined clusters ranging from 0.941 to 1.278 Å (Table 4; Supplementary Material). The superposition of the crystal structure with the different MD clusters shows there is a bad agreement when the MD simulations are performed in the presence of a Mg^2+^ ion and a better agreement when this is absent, and the ionic strength increases. (Table 4; Supplementary Material). In Figure 5, there is a comparison between the LytR LCP domain crystal structure and the models representing the most populated clusters for MD1, MD2 and MD3.

**FIGURE 6:**
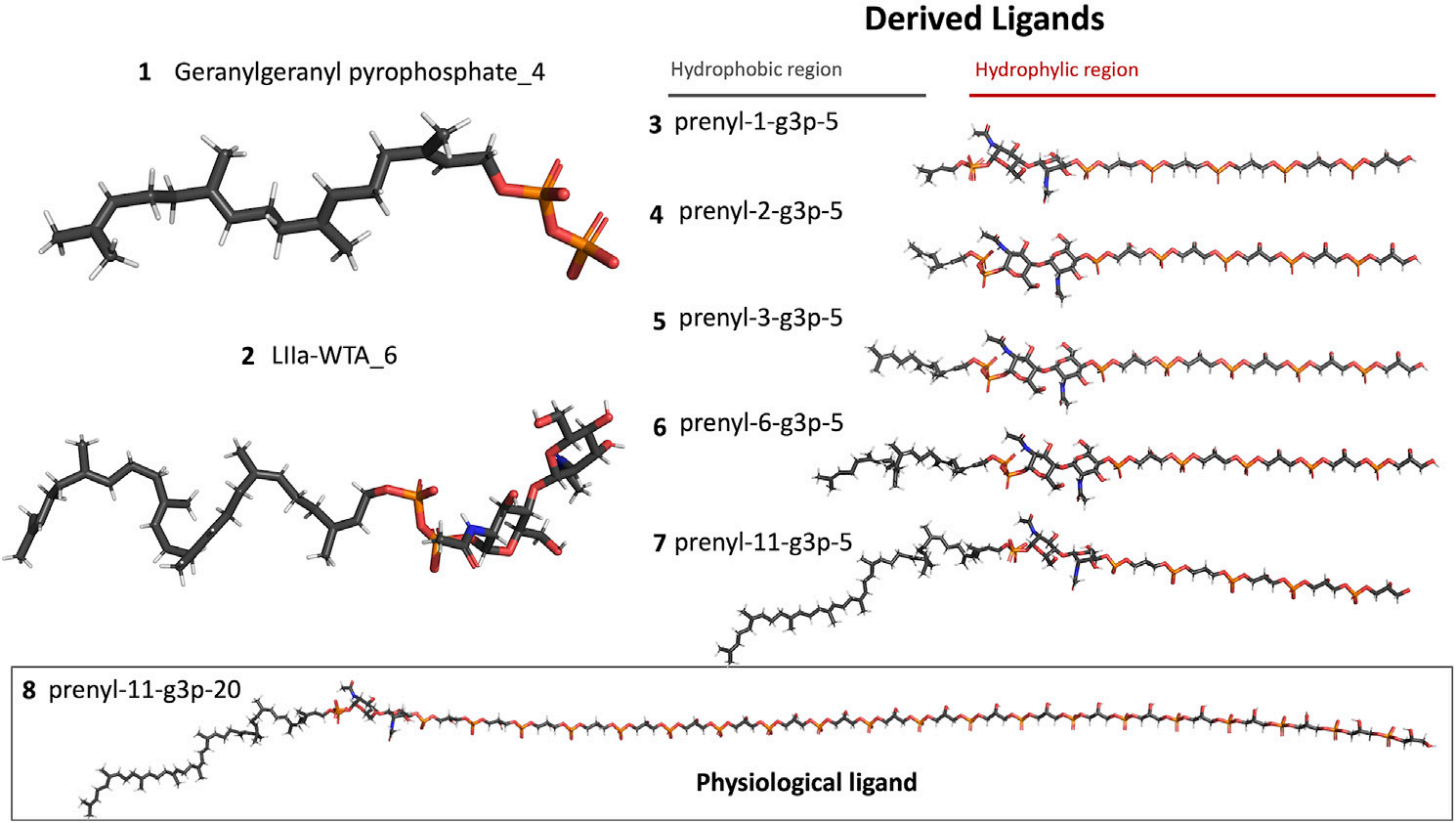
Representation of the different ligands used in the molecular dynamic’s simulations. The ligand in the box, prenyl-11-g3p-20, represents the ligand that more resembles the native substrate of the LCP proteins (MD data not included for this ligand).

**FIGURE 7:**
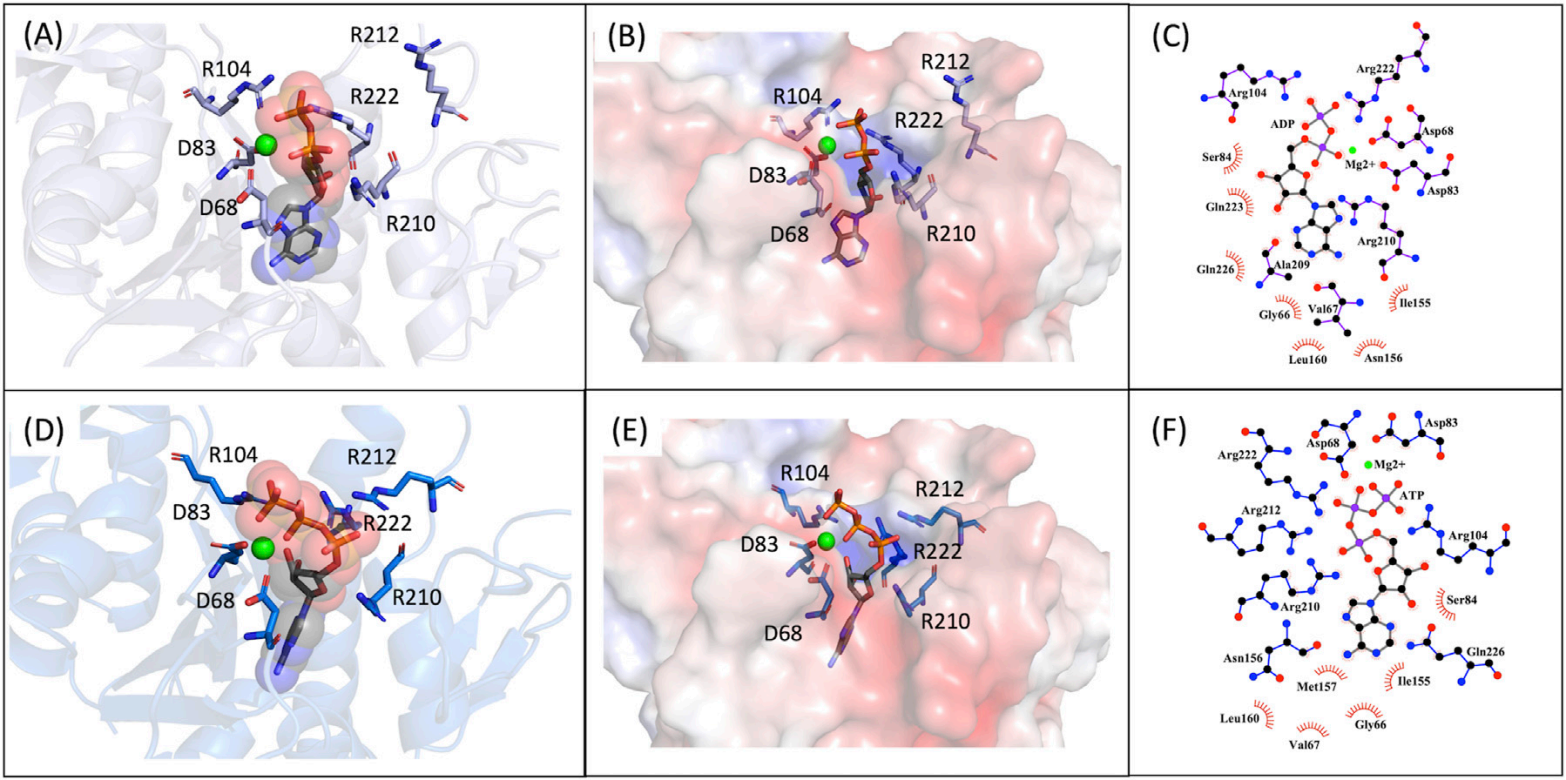
MD9 and MD10 representative structures of the most populated clusters. **(A)-** Cartoon representation of the MD9 representative structure of the most populated cluster, the conserved aspartate and arginine residues are represented as sticks (in purple), the ADP is represented as sticks and spheres with transparency (in grey), and the Mg^2+^ ion is represented as a sphere (in green). **(B)-** Electrostatic surface of the MD9 representative structure of the most populated cluster, the conserved aspartate and arginine residues and the ADP are represented as sticks (in purple and in grey, respectively), and the Mg^2+^ ion is represented as a sphere (in green). **(C)-** 2D representation of the interacting residues with ADP. The ADP (in grey) and the residues performing hydrogen bonds (in purple) are represented as ball and stick and the ones performing hydrophobic interactions represented by the label and red mark. **(D)-** Cartoon representation of the MD10 representative structure of the most populated cluster, the conserved aspartate and arginine residues are represented as sticks (in purple), the ATP is represented as sticks and spheres with transparency (in grey), and the Mg^2+^ ion is represented as a sphere (in green). **(E)-** Electrostatic surface of the MD10 representative structure of the most populated cluster, the conserved aspartate and arginine residues and the ATP are represented as sticks (in purple and in grey, respectively), and the Mg^2+^ ion is represented as a sphere (in green). **(F)-** 2D representation of the interacting residues with ATP. The ATP (in grey) and the residues performing hydrogen bonds (in blue) are represented as ball and stick and the ones performing hydrophobic interactions represented by the label and red mark.

We also carried out MD simulations in the presence of a Mg^2+^ ion and different ligands (Figure 6, molecules 1 and 2), inspired by the physiological substrates of LCP proteins (Figure 6, molecule 8). The results obtained suggest that the length of the hydrophobic region does not impact the binding and that the determinant for the protein-ligand interaction is the presence of the pyrophosphate group. To test this hypothesis, we designed several other ligands with different numbers of prenyl units in the hydrophobic chain (corresponding to molecules 3 – 7 in Figure 6) with the smallest chain having only one prenyl unit. In all cases, the ligands kept bound to the protein during the entire simulation. As expected, the WTA part of the precursor stayed outside the pocket, with a wide range of movement of the glycerol-3-phosphate moieties, that interacted non-specifically with the protein. We also performed MD simulations in the presence of ADP presuming that this could be a putative hydrolysable substrate of the LCP domains. The results suggest that, not only the diphosphate group is responsible for keeping the ADP bound to the protein, allowing the molecule to remain in the active site during the entire simulation, but also that the binding mode is very different from the physiological substrates. According to the structures of TagT from *B. subtilis* (6MPS and 6MPT) and LcpA from *S. aureus* (6UEX), that was crystallized in the presence of glycopolymers, we were expecting to find the sugar moiety of the ligands sitting outside the pocket. However, in the case of ADP MD simulation, the sugar and purine moieties are held inside the pocket. Most likely, the lack of a long hydrophobic chain occupying the hydrophobic pocket can help explain this behavior. Additionally, an MD simulation was performed in the presence of a Mg^2+^ ion and ATP to evaluate if it would compete for the same binding site as ADP. Figure 7 shows the binding modes for the two molecules of the representative structures of the most populated cluster from each MD simulation. The results show a similar binding mode for both molecules, in the active site. However, ATP interacts with the Mg^2+^ ion through the β- and γ-phosphates, whereas the α-phosphate interacts only with R210 and R212. This suggests that LytR LCP domain can cleave the bond between the β- and γ-phosphates, converting ATP to ADP and subsequently ADP into AMP. These results support the hypothesis of competition of ADP and ATP for the active site, as well as the ability of the protein to hydrolyze both molecules.

The R.M.S.D. and R_g_ plots of the molecular dynamics simulations (MD1 - MD10) are shown in Supplementary Material, Figures 2–4.

### 3.4 Docking

Docking was employed to study the interaction of the LytR LCP domain with ADP and compare its binding affinity with known protein-binders (LIIa-WTA, LI-WTA, octaprenyl-pyrophosphate-GlcNAc and octaprenyl diphosphate) that were used as validation molecules (Kawai et al., 2011; Schaefer et al., 2018). Fisetin and ellagic acid, although known anti-biofilm agents, were also used as validation molecules, but as negative controls, as a thermal shift assay (TSA) was conducted that showed no evidence of binding (data not included) Supplementary Figure S5 shows the binding modes predicted by the scoring functions PLP, ASP and Goldscore, for the validation molecules (binders and non-binders). All the scoring functions present in Gold were tested and the respective scores and normalized scores are present in Table 4.

All the scoring functions can correctly discriminate the known protein binders from the non-binders, both in terms of absolute scores and relative scores. For the known protein binders, the scores for PLP ranged from 133.59 to 152.52 and from 2.28 to 2.73, for ASP from 41.61 to 55.57 and from 0.69 to 0.91, for Chemscore from 45.67 to 50.19 and from 0.68 to 1.01, and for Goldscore from 110.80 to 127.26 and from 1.71 to 2.53, absolute and relative values, respectively. For the known non-binders, the scores for PLP ranged from 44.83 to 63.41 and from 2.04 to 3.02, for ASP from 22.63 to 30.05 and from 1.03 to 1.43, for Chemscore from 22.14 to 27.44 and from 1.01 to 1.31 and for Goldscore from 53.83 to 59.46 and from 2.45 to 2.83, absolute and relative values, respectively.

Apart from Chemscore, all scoring functions estimate an absolute score for ADP well above the negative controls, and close to the positive controls, even considering its much smaller molecular weight. When normalizing the score of ADP by the number of heavy atoms, and comparing with the positive controls, the resulting scores with PLP, ASP and Goldscore are significantly higher than those of the positive controls, suggesting the ADP exhibits a higher relative binding affinity than the positive controls taking its size into account.

In Table 4, we can observe that in all cases the scores for the known protein binders are higher than for the known non-binders. In the case of ASP, that difference is less pronounced, yet all the known binders have higher scores than the non-binders. Regarding the values normalized by heavy atoms, the non-binders always present higher scores than the known binders. As the non-binders are smaller molecules this explains the difference between the absolute and relative scores. Regarding ADP we see that, besides Chemscore, the normalized values are always close to the non-binders but slightly higher. As both non-binders and ADP have similar sizes, it is possible to directly compare realizing that ADP has a higher binding affinity than the non-binders, suggesting that ADP can interact with the LytR LCP domain. We used molecular docking to further evaluate and support the hypothesis of competition between ADP and ATP. The scores for ADP and ATP are very similar for all the scoring functions, with ATP having slightly higher scores, suggesting similar affinities from both molecules towards LytR LCP domain. This result supports the hypothesis of competition between the two molecules for the active site. It also suggests that the presence of a third phosphate does not seem to strongly increase the affinity towards the LytR LCP domain.

**FIGURE 8:**
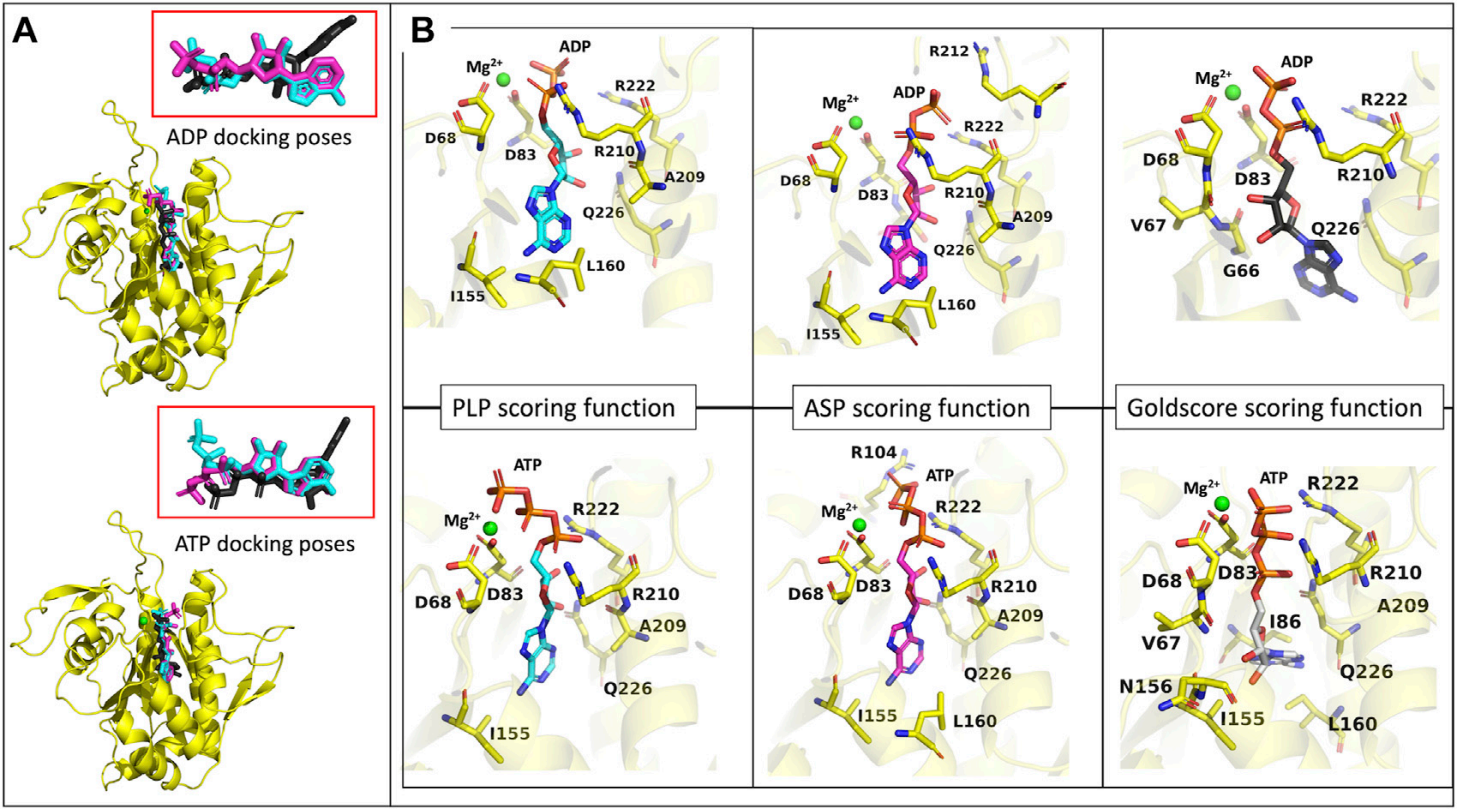
ADP and ATP binding modes predicted by the GOLD three best scoring functions. **(A)**- representation of the model representing the most populated cluster of MD3 (yellow), in the presence of a Mg^2+^ ion (green), with the superposition of the binding modes of ADP and ATP, predicted by PLP (light blue), ASP (pink) and Goldscore (black) scoring functions. Ampliation of the binding modes alone for both molecules. **(B)**- representation of the binding modes predicted by the three best scoring functions, for ADP and ATP, the Mg^2+^ ion (green) and the amino acids important for interaction.

Figure 8 highlights the binding modes predicted by the three best scoring functions for ADP and ATP. The superposition of the models shows that the binding modes are very similar for the PLP and ASP scoring functions, with slight changes in the position of the diphosphate group and the sugar moiety. Regarding the Goldscore, the predicted binding mode presents slight changes in the position of the diphosphate group and pronounced differences in the position of the purine moiety. Interestingly, in the case of ATP, despite the resemblance of the binding poses, PLP and ASP predict more exposed poses, when comparing with ADP, while Goldscore predicts a more buried one.

To analyze the specific interactions between the ligands and the protein amino acid residues, we divide them into three groups: the interactions with (i) the phosphates, (ii) the purine moiety, and (iii) the sugar moiety (See Figure 8). Regarding the first (i), the three best scoring functions predict the interaction between the Mg^2+^ ion and the α- and β-phosphates for ADP, and β- and γ-phosphates for ATP. The phosphates are also involved in salt bridges with and the arginine residues R210 and R222. The consistency of these two arginines in forming ionic interactions with the phosphates, irrespective of the number of phosphates or scoring function, highlights their significance. Additionally, ASP scoring function suggests further interactions with R212 for ADP, and R104 for ATP. The interactions between the protein active site and the purine moiety (ii) are hydrophobic and through hydrogen bonds. In the case of ADP, the different scoring functions predict hydrophobic interactions with I155, L160, and Q226. In the case of ATP, because this molecule is not as buried in the pocket as the diphosphate counterpart, the PLP scoring function does not predict any hydrophobic interactions, while the other scoring functions predict interaction with L160, I86, I230 and Q226. The interaction with I86 further elucidates the more buried pose predicted by Goldscore for ATP since this residue is located inside the substrate binding pocket. Regarding hydrogen bonds, the N6 of the purine moiety is hydrogen bonded to the protein, either to I155 in both ATP and ADP, or to L160 for ADP or Q226 for ATP. The interactions with the sugar moiety (iii) are done through hydrogen bonds with another set of residues. For ADP and ATP, the interacting residues that are common for most of the scoring functions are A209, R210 and Q226. G66 and V67 have also been predicted for ADP interaction, and V67, I155 and N156, for ATP.

The hydrophobic interactions and hydrogen bonds with the purine moiety and the hydrogen bonds with the sugar moiety help elucidate why the predicted binding modes of the two molecules position these parts of the ligands inside the pocket. In contrast, physiological substrates interact with the sugar moiety at the surface. These findings suggest the potential to design competitive inhibitors featuring two or three phosphate groups that interact with the catalytic residues, along with an amphipathic region occupying the elongated substrate pocket. Additionally, the protein’s flexibility may facilitate the entry into the pocket of long hydrophobic tails, as in the physiological substrates, and bulkier ligands as in ADP/ATP.

### 3.5 Activity assays

We used the malachite green assay to determine if ADP and ATP could be hydrolysed by LytR LCP domain, by measuring the release of inorganic phosphate (Pi) and the formation of AMP or ADP. In the presence of ADP, the protein was able to release 110 µM of Pi, corresponding to a conversion of 14.7% of the substrate, whereas in the presence of ATP it was ablet to release 435 μM, corresponding to a conversion of 29%. This result indicates that, under these conditions, not only LytR LCP domain is properly folded and active but is also apt to hydrolyze ADP or ATP, corroborating the role of LCP proteins as pyrophosphatases. Controls where only the ligands or the protein were present did not show an increase in the amount of Pi (Figure 6 from Supplementary Material).

## 4 Discussion

The proteins responsible for the transfer of WTA to the peptidoglycan have remained poorly studied for a long time. In recent years, it has been shown that the LCP family of proteins is involved in cell wall maturation (Kawai et al., 2011). The function of these proteins was confirmed when biochemical assays revealed their capability to transfer the disaccharide present in the linkage unit of the WTAs from lipid-linked precursors to the peptidoglycan (Gale et al., 2017).

In this study, the structure of the LCP domain of LytR protein from *S. dysgalactiae subsp. dysgalactiae* was determined at 2.80 Å, confirming that despite low sequence homology with other LCP-family members, the overall structure is highly conserved.

**FIGURE 9:**
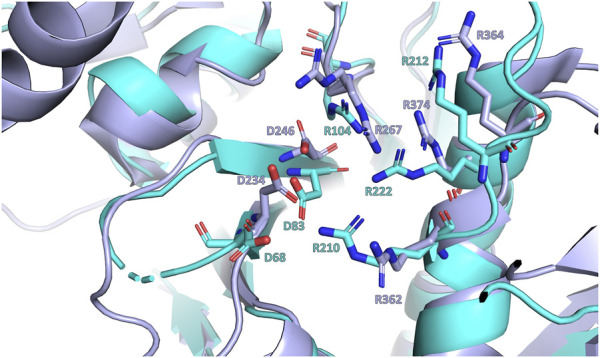
Close-up view of the superposition of LytR (represented in light blue) and Wzg (represented in purple) LCP domains crystal structures (Kawai et al., 2011). The conserved aspartate and arginine residues are represented as sticks.

The presence of the negatively charged residues at the active site, typically two aspartic residues, is crucial for the protein’s activity. In 2011, Kawai and collaborators showed that a D234 A mutation of the protein Wzg from *S. pneumoniae* significantly reduced the protein activity. The same result was obtained in the presence of a chelating agent (EDTA), suggesting that the Mg^2+^ ion is also required for catalysis (Kawai et al., 2011). More recently, studies by Gale et al. and Schaefer et al., using LCP proteins from *B. subtili*s and *S. aureus*, respectively, demonstrated that the transfer of WTAs to the peptidoglycan is performed by LCP proteins (Gale et al., 2017; Schaefer et al., 2018). As expected, the assays conducted in the presence of EDTA inhibited the transfer. The active site is also decorated with four conserved arginies, and to assess their role, the authors prepared the single mutant R267A and observed that the protein’s ability to interact with a pyrophosphate-containing substrate was unaffected, compared to the wild-type (Kawai et al., 2011). This observation is further validated by deposited structures of this mutant with the ligand in the hydrophobic pocket. Schaefer and colleagues studied the impact of the mutations R118A, R219A and R227A on the TagT protein from *B. subtilis* for transferring the LIIa-WTA to the peptidoglycan. The authors observed that each single mutation in the arginine residue completely abolished the protein’s transfer reaction. The results highlight the importance of the arginine(s) for activity but not so much for the interaction with the ligand. In the superposition of LytR and Wzg, these aspartates and arginine residues occupy the same positions in LytR, suggesting a similar role for the activity of this protein (Figure 9).

To the best of our knowledge, all deposited crystal structures, regardless of the presence or absence of ligands, show a compact conformation. This conformation is likely easier to crystallize. However, the ability of the protein to adopt different conformations should not be excluded as it might be associated with its physiological role. During catalysis, the protein accommodates long aliphatic chains inside the hydrophobic pocket which are released after delivering the WTA acid to the peptidoglycan. A more relaxed and open conformation might facilitate substrate binding and product release, with the protein adopting a more closed conformation during the cleavage of the diphosphate bond and the formation of the new phosphate bond. The SAXS experiments performed in the presence and absence of ligand support this hypothesis; the presence of geranylgeranyl-pyrophosphate induces a closed conformation, whereas its absence promotes a relaxed form.

Molecular dynamics simulations were conducted with various ligands, including physiological substrates, ADP, and ATP, to explore their interactions with the protein. The findings highlighted the pivotal role of the pyrophosphate group in ligand binding, whereas the length of the hydrophobic region was less influential. Distinctive binding behaviors were observed for ADP and ATP compared to physiological substrates, with these nucleotides positioning their sugar and purine components within a lengthy and narrow pocket. These simulation results were corroborated by experimental evidence using the malachite green assay, which confirmed the protein’s capacity to cleave the pyrophosphate bonds in ADP and ATP, releasing inorganic phosphate. Additionally, docking studies reinforced these observations, indicating that ADP and ATP could compete with physiological substrates for the active site in the LytR LCP domain. Although ADP and ATP showed lower absolute binding scores relative to known binders, their normalized scores indicated they are still competitively bound. Notably, the extra phosphate in ATP did not substantially enhance its affinity, with only the β- and γ-phosphate groups showing significant interactions. Given their hydrophilic properties and ease of handling in solutions, ADP and ATP are recommended as primary substrates in enzymatic assays for upcoming drug development initiatives. Furthermore, these results open new pathways for creating inhibitors targeting this protein family without requiring long hydrophobic tails.

Taken together, the structural and function data of the LCP domain of LytR protein from *S. dysgalactiae subsp. dysgalactiae* offer promising new directions for therapeutic strategies against bacterial infections.

## Data Availability

The datasets presented in this study can be found in online repositories. The names of the repository/repositories and accession number(s) can be found below: https://www.rcsb.org/, 8QTY; https://www.sasbdb.org/, SASDTH2; https://www.sasbdb.org/, SASDTG2.

## In memoriam

In memory of Ilda Santos-Sanches, an excellent and inspiring teacher.
