# Supplementary figures and images for: Structural insights of an LCP protein–LytR–from Streptococcus dysgalactiae subs. dysgalactiae through biophysical and in silico methods
Source: Front Chem. 2024 Aug 6;12:1379914. doi: 10.3389/fchem.2024.1379914 (PMC11337229; doi:10.3389/fchem.2024.1379914)

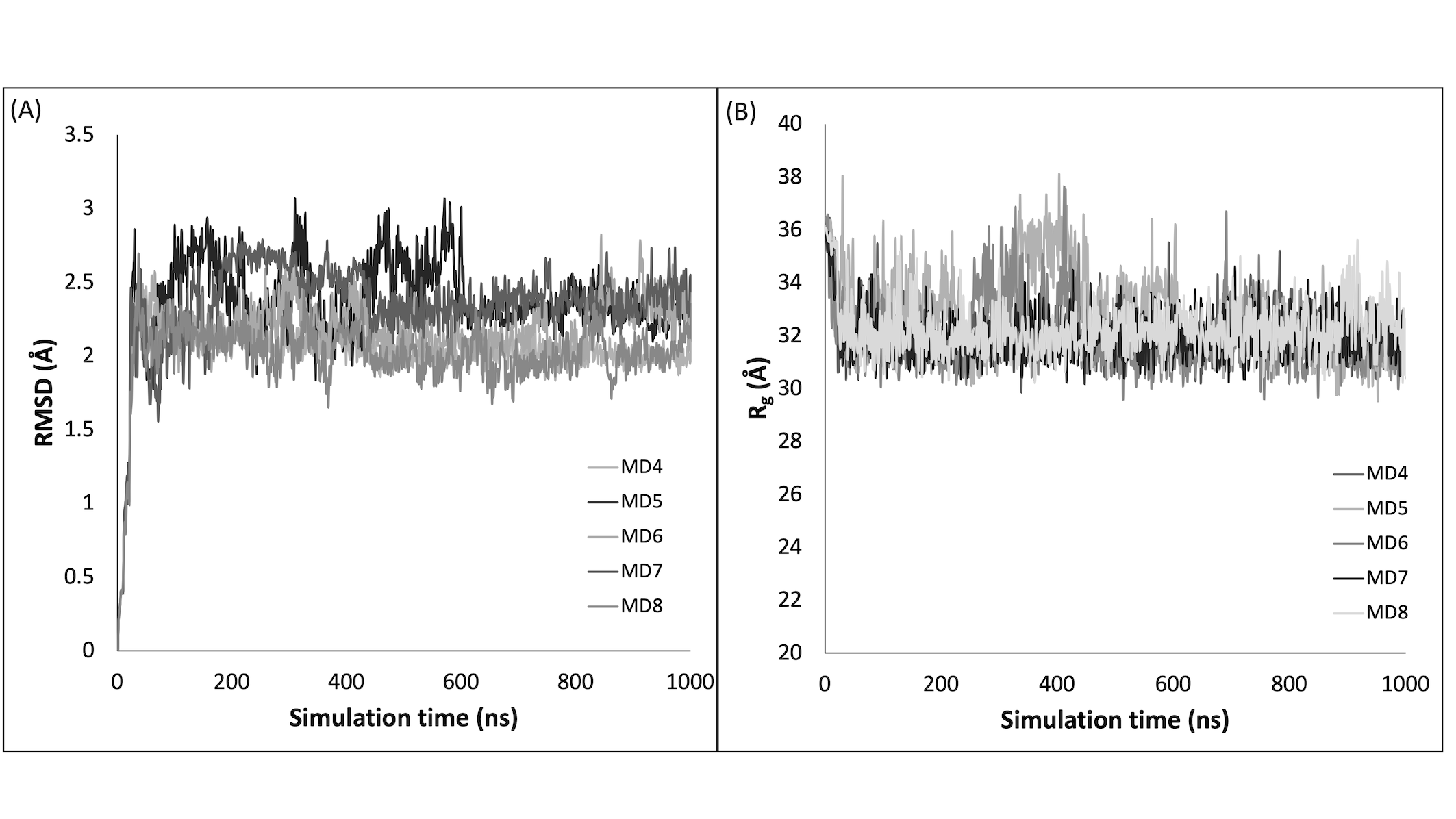

Supplement: Supplementary file 1 [file Presentation1.ZIP › Supplementary Material Presentation/Suppl.Figure_3.tiff]

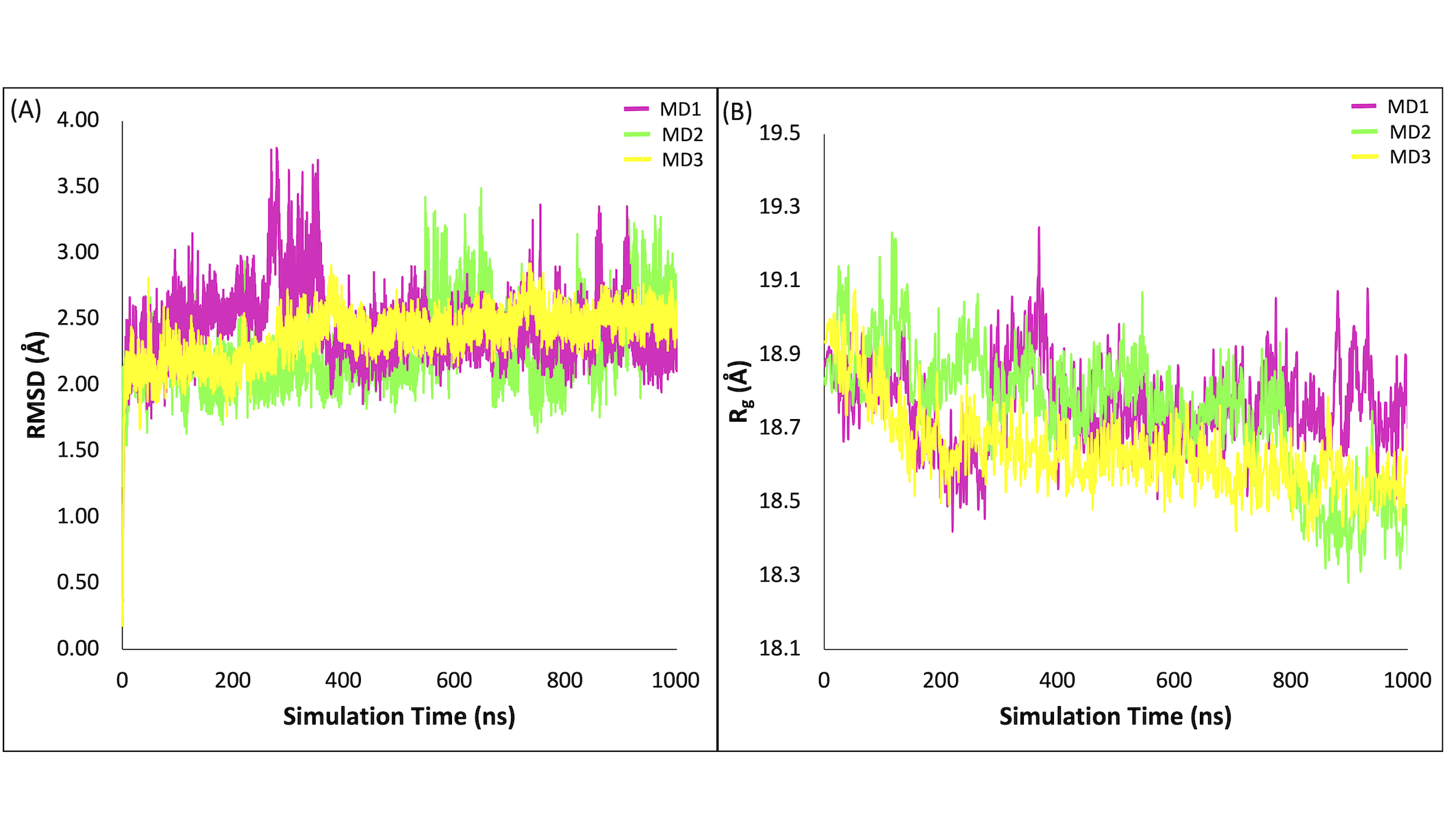

Supplement: Supplementary file 1 [file Presentation1.ZIP › Supplementary Material Presentation/Suppl.Figure_2.tiff]

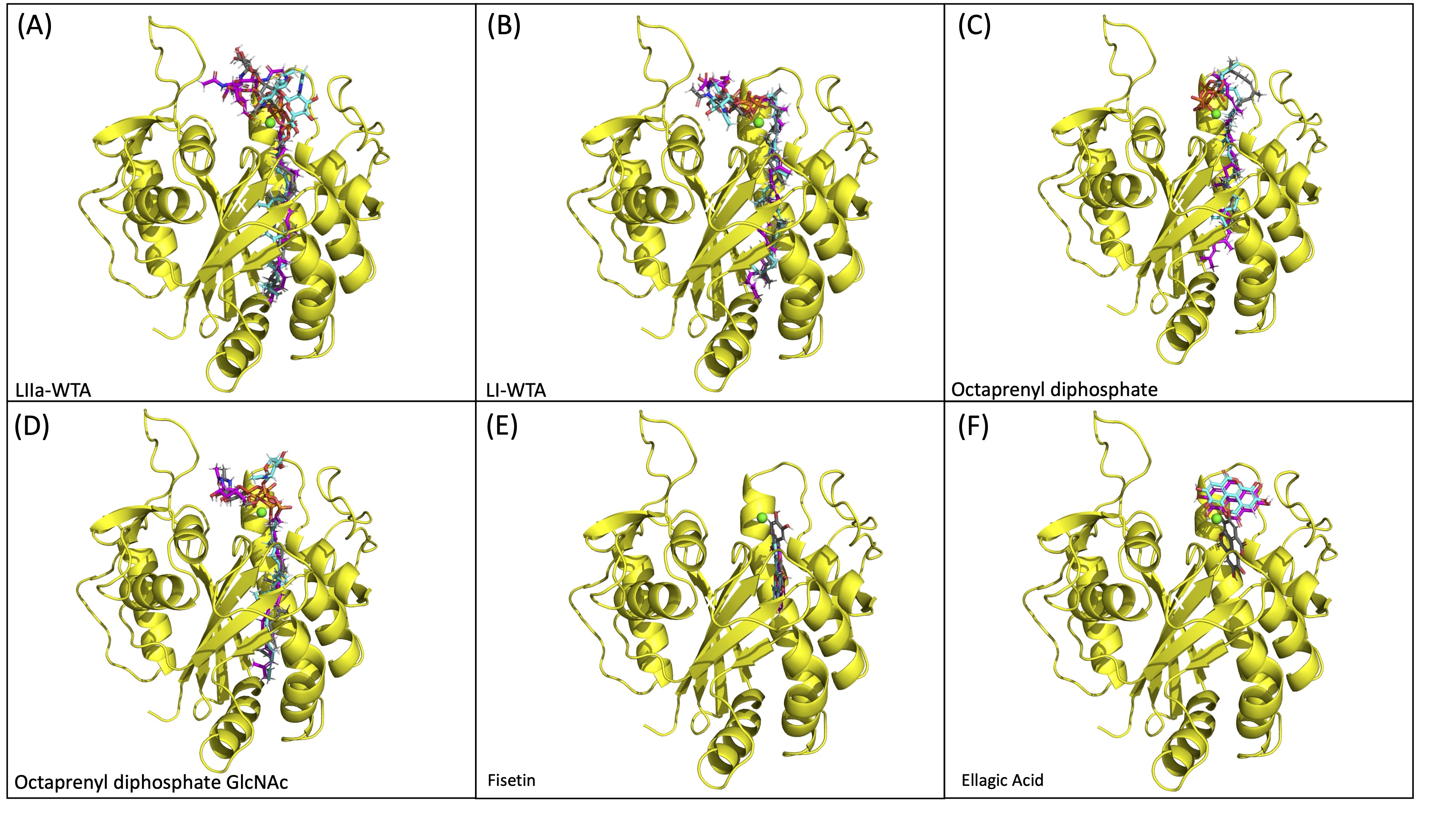

Supplement: Supplementary file 1 [file Presentation1.ZIP › Supplementary Material Presentation/Suppl.Figure_5.tiff]

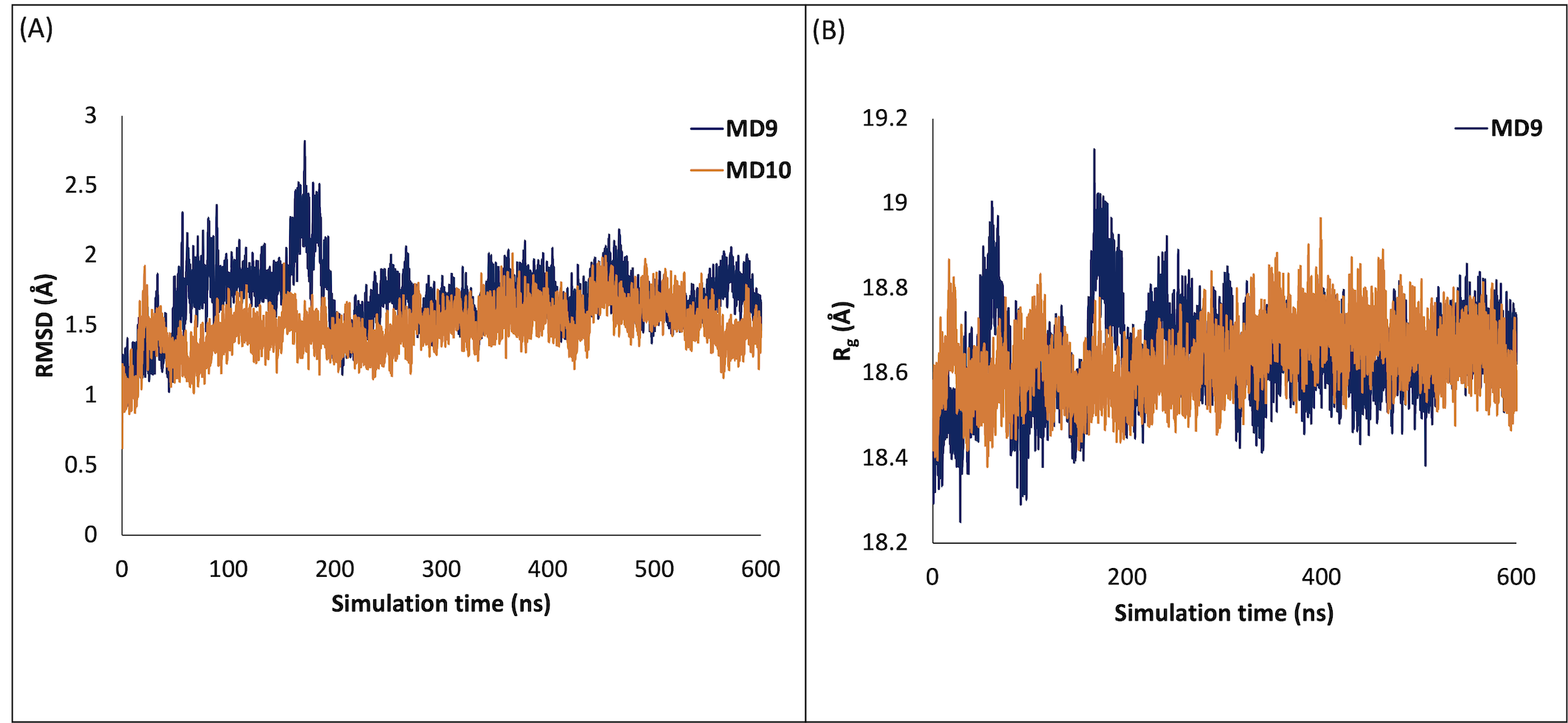

Supplement: Supplementary file 1 [file Presentation1.ZIP › Supplementary Material Presentation/Suppl.Figure_4.tiff]

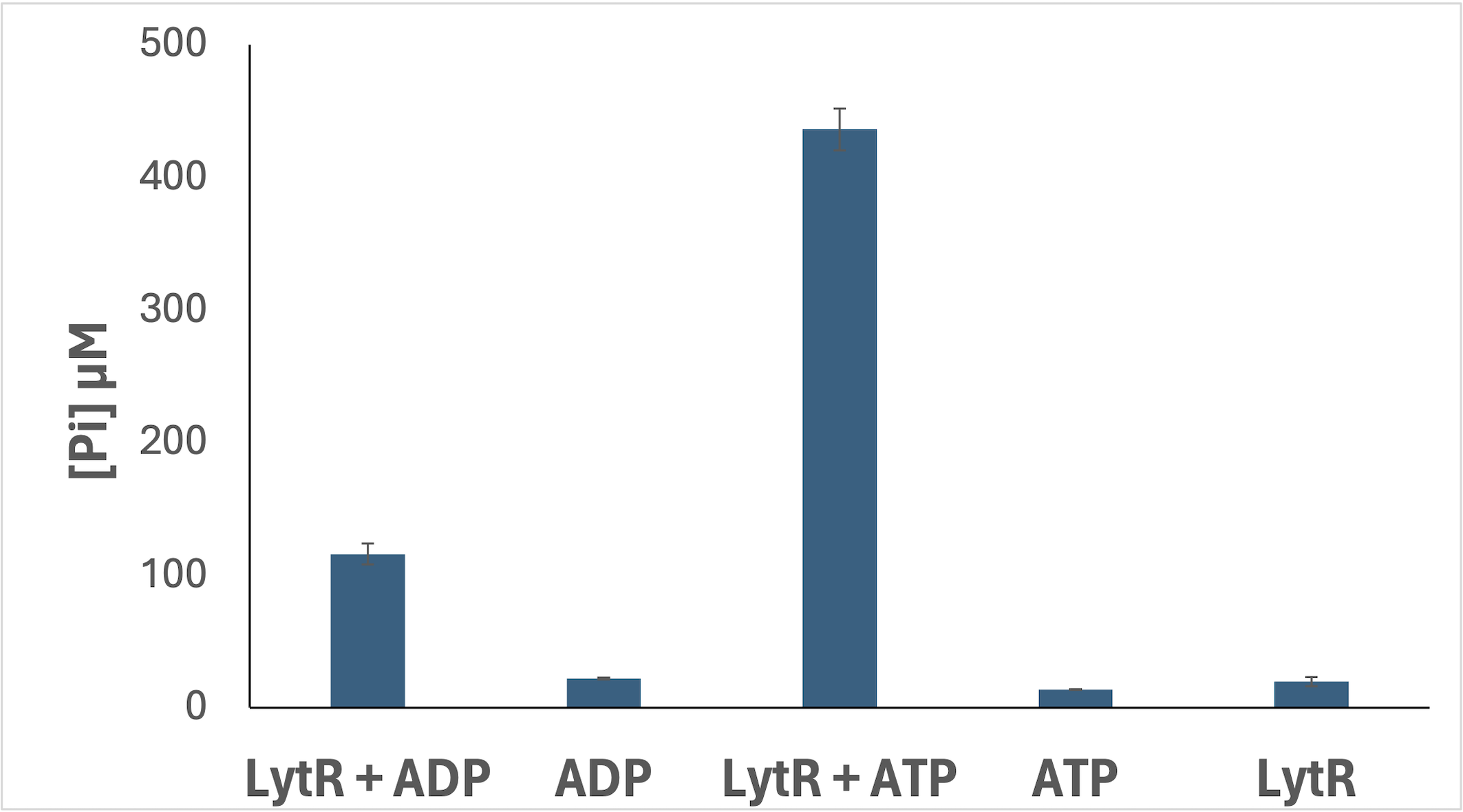

Supplement: Supplementary file 1 [file Presentation1.ZIP › Supplementary Material Presentation/Suppl.Figure_6.tiff]

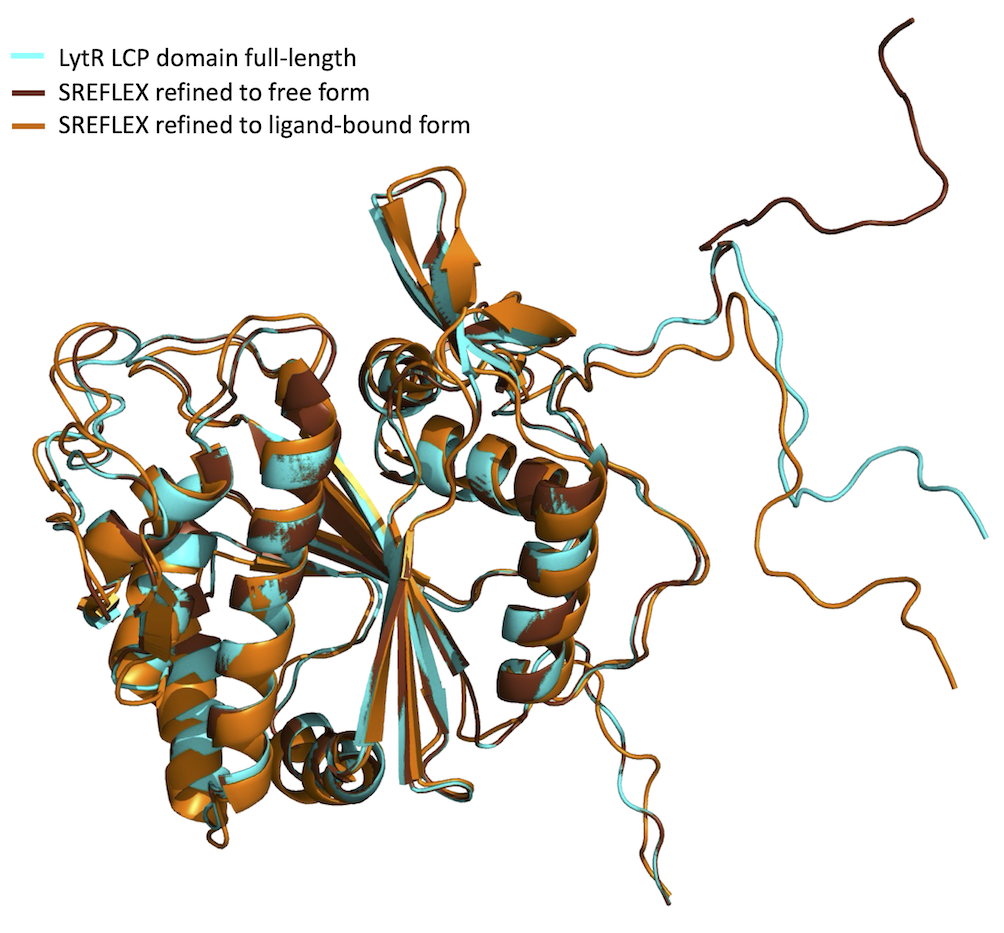

Supplement: Supplementary file 1 [file Presentation1.ZIP › Supplementary Material Presentation/Suppl.Figure_1.tiff]
