# Supplementary material for: Structural insights of an LCP protein–LytR–from Streptococcus dysgalactiae subs. dysgalactiae through biophysical and in silico methods
Source: Front Chem. 2024 Aug 6;12:1379914. doi: 10.3389/fchem.2024.1379914 (PMC11337229; doi:10.3389/fchem.2024.1379914)
Supplement: Supplementary file 2 [file DataSheet1.docx]

Supplementary Material

# Supplementary Data

# Supplementary Figures and Tables

## Supplementary Figures

Figure 1 – Comparison between the LCP domain full-length (light blue), and the refined models from SREFLEX to the free form data (brown) and ligand-bound forms (orange).

Figure 2 – (A)- RMSD variation over the molecular dynamic’s simulation time for MD1 (pink), MD2 (green) and MD3 (yellow). (B)- R_g_ variation over the molecular simulations time for MD1 (pink), MD2 (green) and MD3 (yellow).

Figure 3 – (A)- RMSD variation over the molecular dynamic’s simulation time for MD4 to MD8. (B)- R_g_ variation over the molecular dynamic’s simulation time for MD4 to MD8.

Figure 4 – (A)- RMSD variation over the molecular dynamic’s simulation time with ADP. (B)- R_g_ variation over the molecular dynamic’s simulation time with ADP.

## Supplementary Tables

Table 1 – Primers used for cloning.

| Primer name | Oligonucleotide (5’-3’) |
| --- | --- |
| Primer forward – Nde1 | TACATATGTCCAAAAAAAGTCATGGGATTAAGCAG |
| Primer reverse - XhoI | GTGCTCGAGTGCGCTCTTTCTTGAGC |

Table 2 – Molecular dynamics simulations conditions

|  | Counter-ions | Water molecules | Conditions | | |
| --- | --- | --- | --- | --- | --- |
|  |  |  | Mg^2+^ | Ionic strength | Ligand |
| MD1 | 3 Na+ ions | 14,834 | 1 ion | No | No |
| MD2 | 5 Na+ ions | 14,574 | No | 500 mM NaCl (130 Na+ and 130 Cl- ions) | No |
| MD3 | 5 Na+ ions | 14,801 | 1 ion | No | LII-WTA |
| MD4 | 10 Na+ ions | 19,394 | 1 ion | No | Prenyl-1-g3p-5 |
| MD5 | 10 Na+ ions | 19,418 | 1 ion | No | Prenyl-2-g3p-5 |
| MD6 | 10 Na+ ions | 19,419 | 1 ion | No | Prenyl-3-g3p-5 |
| MD7 | 10 Na+ ions | 19,420 | 1 ion | No | Prenyl-6-g3p-5 |
| MD8 | 10 Na+ ions | 19,420 | 1 ion | No | Prenyl-11-g3p-5 |
| MD9 | 6 Na+ ions | 13,955 | 1 ion | No | ADP |

Table 3 – *S. pneumoniae* Wzg structures comparison between wildtype and R267A mutant and in the presence of decaprenyl phosphate and octaprenyl diphosphate

| Wzg structures IDs | Ligands | RMSD (Å) |
| --- | --- | --- |
| 2XXP (WT) vs 4DE8 (R267A) | Decaprenyl phosphate | 0.14  (321 aligned Cα out of 380) |
| 3TFL (WT) vs 2XXQ (R267A) | Octaprenyl diphosphate | 0.20  (360 aligned Cα out of 374) |
| WT - 2XXP vs 3TFL | Decaprenyl phosphate vs Octaprenyl diphosphate | 0.38  (341 aligned Cα out of 372) |
| R267A - 4DE8 vs 2XXQ | Decaprenyl phosphate vs Octaprenyl diphosphate | 0.38  (310 aligned Cα out of 374) |

Table 4 – Comparison of the representing models of each cluster for MD1 and MD2 and against LytR LCP domain

| MD model | Cluster (frequency, %) | vs Cluster (Å) | | | vs LytR LCP domain (Å) |
| --- | --- | --- | --- | --- | --- |
|  |  | 1 | 2 | 3 |  |
| MD1 | 1 (87.6) | - | 0.758  240/276 | 1.245  268/276 | 1.287  236/268 |
|  | 2 (10.6) | - | - | 1.491  262/276 | 1.298  248/268 |
|  | 3 (1.6) | - | - | - | 2.078  246/268 |
| MD2 | 1 (40.6) | - | 1.278  224/276 | 0.941  223/276 | 0.951  219/268 |
|  | 2 (37.1) | - | - | 1.188  218/276 | 0.948  210/268 |
|  | 3 (22.3) | - | - | - | 0.926  217/268 |
